# Helicobacter Pylori-induced BRD2 m^6^A modification sensitizes gastric cancer cells to chemotherapy by breaking FLIP/Caspase-8 homeostasis

**DOI:** 10.7150/ijbs.97464

**Published:** 2025-01-01

**Authors:** Sen Wang, Zhe Xuan, Zetian Chen, Penghui Xu, Lang Fang, Zheng Li, Yigang Zhang, Hongda Liu, Linjun Wang, Diancai Zhang, Hao Xu, Li Yang, Zekuan Xu

**Affiliations:** 1 Gastric Cancer Center, Department of General Surgery, The First Affiliated Hospital of Nanjing Medical University, Nanjing, China.; 2 Institute of Gastric Cancer, Nanjing Medical University, Nanjing, China.

**Keywords:** *H. pylori*, BRD2, DNA damage, apoptosis, pyroptosis

## Abstract

**Background:** Chemoresistance severely deteriorates the prognosis of advanced gastric cancer (GC) patients. Several studies demonstrated that *H. pylori* (HP)-positive GC patients showed better outcomes after receiving chemotherapy than HP-negative ones. This study aims to confirm the role of HP in GC chemotherapy and to study the underlying mechanisms.

**Methods:** The HP infection co-culture with GC cell lines were performed. The m^6^A-seq and NGS were used for bioinformatic analysis. Western Blot, qRT-PCR and IHC were adopted for expressions of METTL3, BRD2 and YTHDF2. The ATPGlow, flow cytometry and IF were used to detect the cell viability, DNA damage, apoptosis and pyroptosis. Luciferase reporter assay and CHIP were applied to explore the mechanisms.

**Results:** The HP infection sensitized GC cells to 5-FU and induced expressions of METTL3 and YTHDF2. The HP infection promoted transcription of METTL3 through NF-κB pathway, therefore promoting the m^6^A modification level. METTL3 induced the m^6^A modification of BRD2 while YTHDF2 promoted the decay of mRNA of BRD2, both of which could promote the apoptosis and pyroptosis induced by 5-FU. In addition, BRD2 regulated the transcription of FLIP by importing FOXO4 into nucleus, thereby inhibiting the activation of Caspase-8, which was considered as the molecular switch of both apoptosis and pyroptosis.

**Conclusions:** HP-induced m^6^A methylation could sensitize gastric cancers to 5-FU with activation of caspase-8 and induced apoptosis and pyroptosis. The Methylated BRD2 activated by NF-κB pathway regulates Caspase-8 by binding to FLIP-promoter FOXO4. This study provides new sights to the HP-positive gastric cancer chemotherapy.

## Introduction

Gastric cancer is the 5^th^ most common malignant tumor in the world, and the number of tumor-related deaths it causes ranks 5^th^ among all malignant tumors 1. China is a nation with a high incidence of gastric cancer, holding the 5^th^ and third positions in terms of incidence and mortality rates among all malignant tumors with approximately 358,700 new cases each year 2. The treatment of gastric cancer mainly includes comprehensive methods of surgery, chemotherapy, immunotherapy, endoscopy and so on. For patients with advanced gastric cancer, the sensitivity to chemotherapy significantly affects the patient's prognosis. Nonetheless, resistance to chemotherapy has emerged as a critical concern in the extensive management of advanced gastric cancer 3, 4. Therefore, elucidating the molecular mechanisms of gastric cancer chemotherapy resistance is of great significance for the treatment of advanced gastric cancer 5.

Helicobacter pylori (*H. pylori*, HP) infection is widely considered a risk factor for gastric cancer 4, 6. Research on *H. pylori* has been going on for a long time. Following Barry Marshall and Robin Warren's discovery and successful cultivation of Helicobacter pylori in 1982 7, many studies have reported the correlation between *H. pylori* and the development of gastric cancer 6, 8-10. However, recent clinical studies have shown that in patients with advanced gastric cancer who undergo chemotherapy, HP-positive patients have better efficacy and prognosis than HP-negative patients 11-14, and HP may be an independent factor affecting prognosis 8, 15. This implies HP's potential role in modulating gastric cancer's responsiveness to chemotherapy medications, yet research is scarce on how HP infection impacts gastric cancer chemotherapy effectiveness, and the associated mechanisms remain unreported.

The effect of HP on gastric cancer cells is a macroscopic process that involves profound changes in the transcriptome and multiple signaling pathways. Epigenetics is one of the primary ways in which HP regulates gastric cancer cells 16, 17, with studies by Peterson *et al.* suggesting that HP can promote DNA methylation and lead to the development of gastric cancer 18. RNA m^6^A methylation is a comparatively novel epigenetic regulatory mechanism that has been shown to be closely related to cell stress states such as infections and UV irradiation 19-22. Our research also indicates a potential link between the level of m^6^A methylation and how malignant tumors respond to chemotherapy, mirroring earlier findings that reduced m^6^A methylation correlates with resistance to chemotherapy in leukemia and cervical cancer 23, 24. The level of m^6^A is jointly regulated by methyltransferases and demethylases, including methyltransferase complexes such as METTL3, METTL14, and WTAP, and demethylases such as FTO and ALΚBH5. Proteins that bind specifically to m^6^A, such as the YTHDF and IGF2BP families, have the ability to engage with m^6^A sites on RNA, contributing to pre-mRNA splicing and the control of RNA stability, among various roles. Consequently, we hypothesize that HP infection may enhance gastric cancer cells' chemosensitivity through m^6^A methylation.

Interestingly, pathways leading to cell death induced by chemotherapy are not singular. Recent studies have shown that chemotherapy, bacterial or viral infections can lead to a novel form of programmed cell death called pyroptosis 25-27. The activation can be triggered by either the traditional Caspase-1 inflammasome or the atypical Caspase-4, -5, or -11 enzymes, which split the crucial pyroptosis protein Gasdermin D 28. The cleaved Gasdermin D can induce the lysis of the cell membrane and release a large amount of inflammatory factors 29. In recent reports, the apoptosis executor protein Caspase-8 was considered as the molecular switch of apoptosis and pyroptosis and in this study, we also found that in addition to enhancing chemotherapy-induced apoptosis, HP might also link to upregulating the level of drug-induced cell pyroptosis by inducing Caspase-8 28, 30-32.

Bromodomain and extra- terminal motif (BET) protein family is a widely studied protein family which includes BRD2 BRD3, BRD4, and BRDT in mammals 33, 34. It is known as a transcription regulator which helps the transcription factor form complexes and could selectively binds to the acetylated site of histone 4 (H4) via its bromodomains. Also, BRD2 has been reported to be involved in the process of apoptosis and programmed cell deaths in malignant tumors 33, 35, 36. In our study, we noticed its uncovered functions in proptosis and might be the key of HP-induced m^6^A methylation. Moreover, its affinity of H4-like transcription factors might play a role in regulating FLICE-inhibitory protein (FLIP). FLIP is a protein that regulates Caspase-8 activity in the cytoplasm. It has the same DED domain as Caspase-8, but lacks enzyme activity. FLIP can competitively bind to FADD (Fas Associated protein with Death Domain) on the cell membrane, hindering the maturation of Caspase-8^37^. Thus, BRD2 disrupts the homeostasis of the FLP/Caspase-8 homeostasis and regulates apoptosis and apoptosis in HP-positive gastric cancer cells.

In general, our team first proposed that the BRD2 m^6^A modification induced by HP infection is closely related to chemotherapy sensitivity, and clarifying its specific mechanism will provide scientific basis for enhancing the chemosensitivity of gastric cancer and improving patient prognosis, which may be of high clinical value.

## Methods

### Cells and reagents

Human gastric cancer cell lines AGS and MKN-45 were obtained from American Tissue Culture Collection (ATCC, Manassas, VA, USA). AGS cells were cultured in F12 with 10% fetal bovine serum (FBS, Invitrogen Life Technologies, Carlsbad, California, USA) and 1% penicillin/streptomycin (GIBCO, Invitrogen Life Technologies). MKN-45 cells were cultured in RPMI 1640 medium (Wisent, Shanghai, China) with 10% fetal bovine serum (FBS) (Wisent, Biocenter, China) and 1% penicillin-streptomycin. All cells were cultured at 37°C in a humid atmosphere containing 5% CO_2_. Recombinant human TNF-α (No. 300-01A, PeproTech, New Jersey, USA) and Bay 11-7082 (No. S2913, Selleckchem, Houston, Texas, USA) were purchased.

### Human samples

All de-identified human tissue samples were collected from GC patients in the Department of General Surgery of the First Affiliated Hospital of Nanjing Medical University. Samples were collected after surgical resection immediately and quickly frozen in liquid nitrogen. Histologic analyzes were performed by two independent pathologists for scoring. Written informed consent was obtained from all patients. All tissue samples were obtained, coded, and de-identified in accordance with the Institutional Review Board-approved protocols of The First Affiliated Hospital of Nanjing Medical University.

### *H. pylori* culture and infection

The wild-type CagA-positive *H. pylori* strains 26695 and SS1 (rodent-adapted) were kindly provided by Professor Guoxin Zhang. The *H. pylori* strains were cultured on trypticase soy agar supplemented with 5% sheep blood (BD Biosciences, Bedford, Massachusetts, USA) for 3 days and sub-cultured onto fresh agar plates 38. The strains were then grown in Brucella broth containing 10% serum and 10 μg/mL vancomycin at 37 °C under 5% CO_2_ overnight. *H. pylori* was added to gastric cells at a multiplicity of infection of 100:1 for co-culture, and cells were harvested at different time points.

### Western blot

Proteins were extracted from cell lysates using standard methods. Proteins were separated by 10% or 12.5% running gel and stacking gel electrophoresis, made of 40% acrylamide/bis solution, SDS, Tris and other components. Proteins were then transferred to the nitrocellulose membranes (Bio-rad, Hercules, California, USA), followed by blocking in 5% Bovine Fraction V (BSA, 9048-46-8, RPI, Mount Prospect, Illinois, USA) for 3h. After hybridization with primary antibodies at 4°C overnight, the membranes were washed and immunoblotted with secondary antibodies. Images were obtained by the Bio-Rad ChemiDoc XRS + System. Primary antibodies were listed in Supplementary Table 1.

### Quantitative real-time PCR

The TRIzol reagent was used to extract total RNA from cells and tumor tissues, and the NanoDrop 2000 (Thermo Fisher Scientific, Waltham, Massachusetts, USA) was used to assess RNA integrity and quantity. High capacity cDNA Reverse Transcription kit (4368814, Applied Biosystems, Foster City, California, USA) was used to reverse transcribe the RNA. Quantitative PCR was performed with the Universal SYBR Green Master kit on the Bio-Rad CFX Connect Real-time System (Bio-rad, Hercules, California, USA) using a 1:5 dilution of cDNA. The experiments were performed in triplicate and the ΔΔC(t) method was used for analysis. The primer sequences are provided in Supplementary Table 2.

### Flow cytometric analysis

The cells were incubated in 6-well plates at the density of 10^6^/well for 24h under the condition of 37 °C and 5% CO_2_. Flow cytometer (Beckman Coulter Life Sciences, California, USA) was employed for analyzing the proportion of apoptosis and ROS level. For the detection of apoptosis, cells were collected and incubated with the PE-Annexin V Apoptosis Detection Kit (BD Biosciences, Becton, NJ, USA). After 30 minutes incubation, the cells were observed. For the evaluation of reactive oxygen species (ROS) levels, the cells were digested and incubated with 10µM H2DCFDA (Thermo Fisher Scientific, Waltham, Massachusetts, USA) for half an hour. The H2DCFDA was prepared in DMSO and diluted to 5 μM in phosphate buffered solution (PBS). The cells were seeded in 24-well plates, the culture medium was removed, and the cells were washed with PBS. Next, the cells were incubated with the H2DCFDA dye at room temperature in the dark for 40 min. After removing the dye, the cells were covered in phenol red-free DMEM and incubated at 37 °C for 15 min for flow cytometric analysis. Data were analyzed by employing FlowJo software.

### Cell viability assay

The AGS and MKN-45 cells were exposed to 20 μM 5-FU for 48 and 72 hours in order to evaluate cell viability. At every interval, CellTiter-Glo Reagent (Promega, Madison, Wisconsin, USA) was introduced into each well of 24-well plates and left to incubate on a shaker at room temperature for 10 minutes. Afterwards, 50 μl of media was moved into 96-well plates. The BMG FLUOstar OPTIMA Microplate Reader (BMG LABTECH, Cary, North Carolina, USA) was utilized to obtain the data for all experiments conducted in triplicate.

### Colony formation

GC cells were seeded in the 6-well plates at the concentration of 1000 cells/well. Cells were then cultured for 2 weeks. The images were captured after dyeing.

### Immunohistochemistry staining

The tissues were immersed in 4% paraformaldehyde and placed in paraffin blocks for safekeeping. Subsequently, the blocks were sliced into sections measuring 5 micrometers in thickness for additional examination. The sections were positioned on glass slides for immunohistochemistry (IHC) and underwent the standard IHC protocol. After being incubated overnight with primary antibodies at 4 °C and then incubated with secondary antibodies for 1 hour at room temperature, chromogen was applied and the images were captured using the All-in-One Fluorescence Microscope (BZ-X700, Keyence, Itasca, IL, USA). The tissue scores were evaluated by a self-reliant pathologist utilizing ImageJ. ImageJ automatically determined the scores, which were subsequently validated by the pathologist. The scores were displayed as comparative levels of expression in relation to neighboring non-cancerous tissues.

### Immunofluorescence

In each well of an 8-chamber slide, 5000 cells were seeded followed by infection with *H. pylori* or other treatments. Following incubation, the cells were immersed in a 4% paraformaldehyde solution for 45 minutes at 4°C, rinsed, and then permeabilized with a permeabilization solution for 3 minutes on ice. Each well was blocked for 20 minutes in the dark using non-immune goat serum (50062Z, Thermo Fisher Scientific, Waltham, Massachusetts, USA). The Phospho-Histone H2A.X (Ser139) (2577s, Cell Signaling Technology), Villin (GT205202, Gene Tech, Shanghai), CEA (GT2020902, Gene Tech, Shanghai) and CK7(GM701802, Gene Tech, Shanghai) were added and the slides were incubated overnight, followed by incubation with secondary antibodies before being exposed to secondary antibodies for 1 hour. Following DAPI staining, the wells were sealed with a cover glass. The secondary antibodies utilized included Alexa Fluor 568 goat anti-mouse IgG (1:400) and Alexa Fluor 488 goat anti-rabbit IgG (1:400).

### Quantification of m^6^A in total RNA by LC-MS

The *H. pylori* strain 26695 was amplified overnight in Brucella broth supplemented with 10% bovine serum and 10μg/mL vancomycin at 37°C under 5% CO_2_ and was subsequently co-cultured with GC cell line AGS at an MOI of 100:1. Following a 6-hour co-culture, AGS cells were harvested and total RNA was extracted using TRIzol reagent. Subsequently, 1 μg of RNA was treated with 2 U of Nuclease P1 at 37°C for 12 hours to convert RNA into individual nucleotides, following by incubating with alkaline phosphatase and ammonium carbonate mixture at 37°C for an additional 2 hours. The obtained RNA solution was diluted 10 times, and the nucleotides were separated and quantified on reverse phase high-performance liquid chromatography. Mass spectrometry in Multiple Reaction Monitoring (MRM) mode was performed on TripleTOF 5600+(AB Sciex, Framingham, USA) to ionize nucleotides, and m^6^A peak areas were calculated based on the retention times.

### Plasmids and transfections

The full-length of human METTL3 and BRD2 were amplified using polymerase chain reaction and was subsequently cloned into the pcDNA3.1 vector through standard molecular cloning techniques. To ensure accuracy, the inserted sequence was further verified by DNA sequencing and the concentration and purity of the plasmid DNA were assessed using spectrophotometric analysis. METTL3-OE and BRD2-OE plasmids were subsequently utilized for transfection and gain-of-function experiments. The siRNA sequences utilized in this study were as follows: siBRD2: 5'-CTGTCTTTGTTGATTCTAACT-3'; siCtrl: 5'-CTCTCAACCCTTTAAATCTGA-3'. siFLIP: 5'-CCUCCUGGAUAGCUUAAGUUU-3'; siCtrl:5'-UUCUCCGAACGUGUCACGUTT-3'.

### Lentivirus-delivered shRNA gene knockdown

Lentiviral vectors were engineered to facilitate the targeted knockdown of METTL3 and BRD2 genes. pGLV3/H1/GFP lentiviral vectors were purchased from GenePharma (Shanghai, China). For the suppression of BRD2, a specific short hairpin RNA (shRNA) sequence was employed: 5'-CTGTCAAGCGGAAGATGGAGAACCGTGATTACCGGGATGCA-3'. Concurrently, the knockdown of METTL3 was accomplished utilizing shMETTL3 sequence: 5'-GCTGCACTTCAGACGAATTATCTCGAGATAATTCGTCTGAAGTGCAGC-3'. The sequences were cloned into the lentiviral vectors and the insert integrity was validated through sequence verification, ensuring the accuracy of the shRNA sequences within the vectors.

### Chromatin immunoprecipitation assays

Chromatin immunoprecipitation assays were performed as previously described 38. In brief, AGS cells were fixed with formaldehyde and treated with nuclear lysis buffer, and the released chromatin was then fragmented by sonication in six cycles of alternating 30 seconds on and off at 40% amplitude. The resulting DNA fragments were sized between 100-1000 base pairs to optimize the pull-down process. The immunoprecipitation process was carried out with NF-κB-p65 antibody (Ser536) (3033S, Cell Signaling Technology, Danvers, MA, USA) with a normal human IgG as control along with A/G magnetic beads. The mixture was incubated at 4 °C overnight and then eluted prepared for downstream analysis. Primers for potential NF-κB-p65 binding sites and potential YTHDF2 binding sites on BRD2 were in Supplementary Table 2.

### *In vivo* animal tumor model

A total of 30 Balb/c nude mice were purchased from Department of Laboratory Animal Center of Nanjing Medical University. Among them, a total of 24 4-week-old mice were randomly divided into four groups, with 6 mice in each group. The AGS cells transfected with shBRD2 were injected subcutaneously into the axilla of the forelimb (5 × 10^6^ cells per mouse) of six mice in the shBRD2 and shBRD2+5-FU group. The 20 μM 5-FU was injected into axilla of the forelimb 10 days after injection of AGS in 5-FU group and 5-FU+shBRD2 groups. The mice from the control group were injected with shControl twice a week for 2 weeks. After 1 month, all mice were sacrificed and tumor weights were measured. A total of 6 TFF1-KO mice (C57BL/6JGpt-Tff1em6Cd5985/GptTff1-KO) purchased from GemPharmatech (Nanjing, Jiangsu, China) were also adopted for further experiments. Among them, 3 mice were orally administrated with SS1 strain (10^9^ CFU/mouse) three times a week for 4 weeks while other 3 mice were treated with broth. After 4 weeks of oral administration, mice were sacrificed for gastric tissues for further analysis. The Nanjing Medical University Ethics Committee approved all animal experiments involved in this study.

### Statistics

Data was presented as mean ± SD in each experiment. The significant difference was considered as P < 0.05 in each experiment. One-way analysis of variance (ANOVA) and Student's t-test were applied.

## Results

### HP infection sensitized GC cells to 5-FU and induced higher apoptosis and pyroptosis

**Figure 1:**
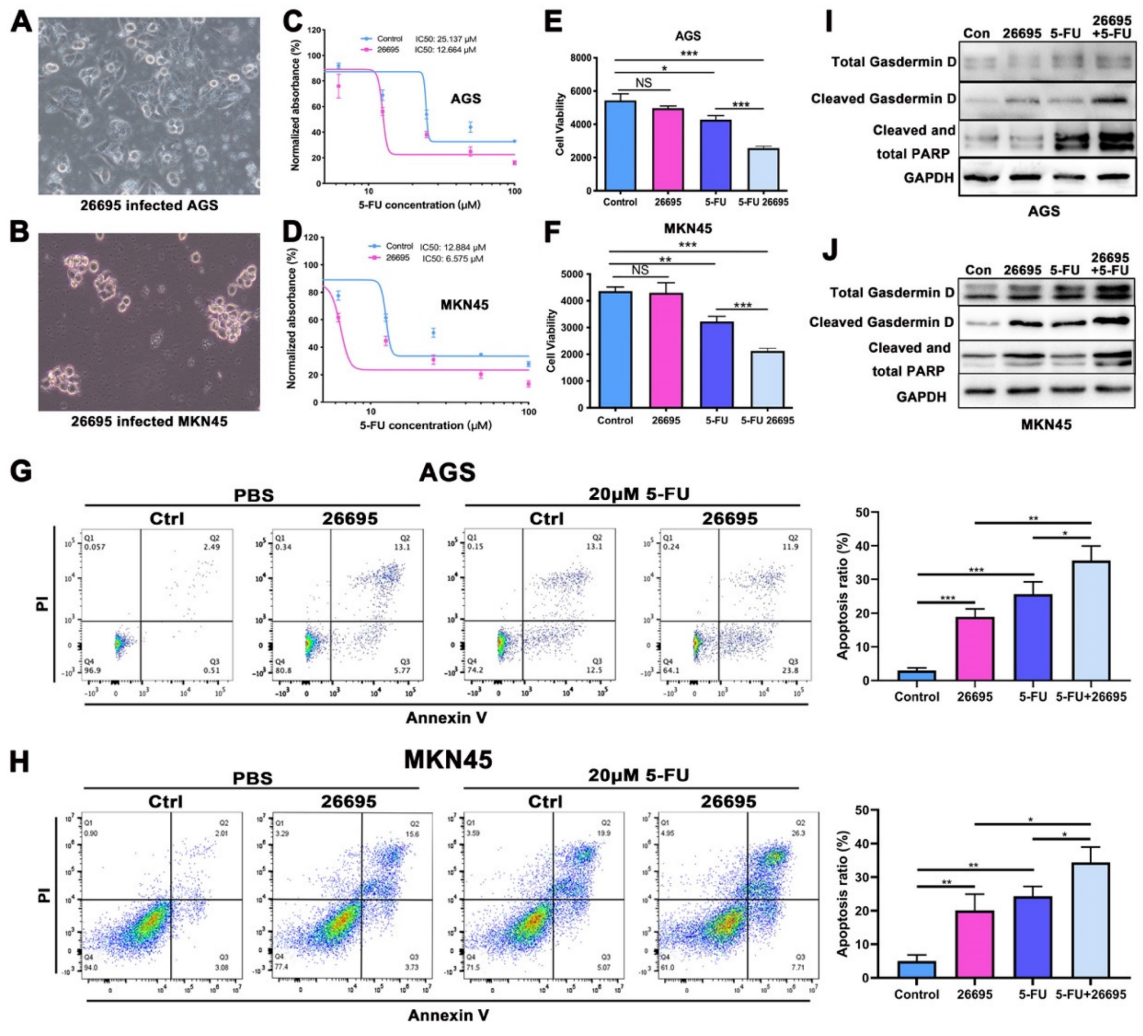
** HP infection sensitized GC cells to 5-FU and induced higher apoptosis and pyroptosis.** (A-B) The AGS and MKN-45 cells were infected with HP strain 26695 after 6h. (C-D) The IC50 assay using ATP-Glow of 5-FU treatment in AGS and MKN-45 cells. (E-F) The ATP-Glow assay in AGS and MKN-45 cells after co-culture of HP strain 26695 and 5-FU treatment. (G-H) The flow cytometry showed the apoptosis of AGS and MKN-45 after co-culture with 5-FU (20 μM) and HP. (I-J) Western blot showed apoptosis and pyroptosis after treatment of 5-FU (20 μM) and HP infection (6h). * P<0.05, ** P<0.01, *** P<0.001.

First we co-cultured HP strain 26695 with AGS and MKN-45 GC cell lines to confirm the HP infection. The images of co-culture of HP and GC cells were taken at 6h after seeding HP into cells (Fig. 1A-B). With 24h of 5-FU and 6h of HP co-culture, cells were harvested for IC50 assay using ATPGlow to detect the sensitivity of 5-FU. The IC50 of 5-FU in AGS and MKN-45 cells for negative control were 25.137 μM and 12.884 μM respectively. While co-cultured with 26695, IC50 was significantly dropped to 12.664 μM and 6.575 μM, respectively (Fig. 1C-D). The cell viability assay validated again that compared to control, HP infection would not significantly affect cell viability in AGS and MKN-45. But after co-culture with 5-FU, HP would significantly decrease the cell viability. After combination of HP co-culture and 5-FU treatment, cell viability dropped most in both cell lines (Fig. 1E-F). These results provided pieces of evidence that HP infection might increase the sensitivity of AGS and MKN-45 to 5-FU.

Since 5-FU is associated with cell DNA damage and cell death, flow cytometry was used then to detect the apoptosis of cells after co-culture with 5-FU and HP (Fig. 1G-H). With staining of PI and Annexin V, we found after co-culture with 26695 for 6h, AGS and MKN-45 both demonstrated more apoptosis than control. With 5-FU treatment, more apoptosis was induced in AGS cells compared to control. Meanwhile, when we used 5-FU treatment and 26695 co-culture for flow cytometry, more apoptosis was induced compared to 5-FU treatment alone. Similar results were obtained from colony formation assays (Figure S1A). This again proved the 26695 strain could enhance apoptosis induced by 5-FU in AGS cells.

As we mentioned above, we found that the 26695 strain could also enhance the comparatively new cell death pathway-pyroptosis, which was induced by 5-FU (Fig. 1I-J). Western Blot showed that after 26695 infection, the pyropotosis marker cleaved Gasdermin D and apoptosis marker cleaved PARP were stronger than control, even also stronger than 5-FU treated alone. However, when cells treated with 5-FU and 26695 together, we found Cleaved Gasdermin D and cleaved PARP was significantly stronger than 5-FU-treated alone, indicating that 26695 may induce higher levels of apoptosis and pyroptosis as well. The immunofluorescence showed that apoptosis-associated speck-like protein (ASC) was also induced most in HP and 5-FU treated groups in both cell lines (Figure S1B).

We also used DNA damage marker p-H2A.X staining to detect the DNA damage of GC cells. As previous results showed, HP treated alone and 5-FU treated alone induced DNA damage compared to control but combining HP treatment and 5-FU treatment, highest density of p-H2A.X was demonstrated in both AGS and MKN-45 cells, indicating highest DNA damage to GC cells (Figure S1C). ROS assay revealed that either HP or 5-FU could promote ROS level in AGS and MKN-45 cells and highest ROS was induced in HP plus 5-FU treatment group (Figure S1D).

We then divided patients underwent radical gastrectomy with p-stage III who received 5-FU chemotherapy into HP+/- groups (17 vs 17) retrospectively and the prognosis of 5-year survival did not demonstrated significant difference (P=0.4601), but it showed the tendency of difference (Figure S1E).

### HP sensitized GC to 5-FU via inducing m^6^A modification

**Figure 2:**
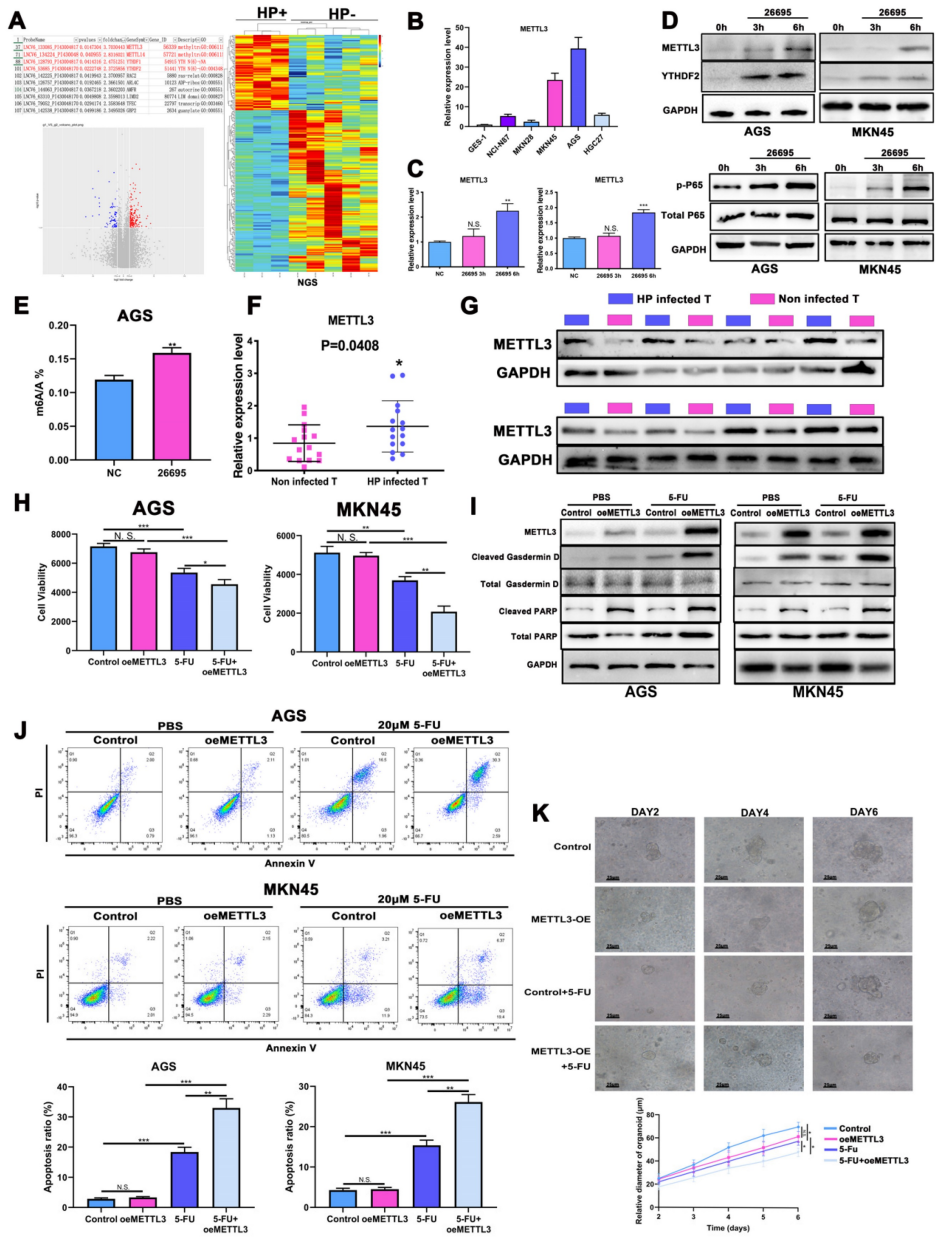
** HP sensitized GC to 5-FU via inducing m^6^A modification.** (A) The RNA-seq data on HP-positive GC tumor tissues with HP-negative GC tumor tissues (3 HP+ vs 5 HP-). (B) The cell expression profiling of METTL3. (C) qRT-PCR analysis confirmed METTL3 expression level was increased after 6h in AGS and MKN-45 cell lines. (D) Western Blot analysis showed that HP infection induced expression level of METTL3, YTHDF2 and p-P65 in AGS and MKN-45 cell lines. (E) LC-MS showed HP infection could induce m^6^A methylation level after 6h. (F) qRT-PCR analysis showed significantly higher expression of METTL3 in HP-infected tumor tissues than non-infected tumor tissues (15 vs 15). (G) WB analysis showed higher expression of METTL3 in HP-infected tumor tissues than in those of non-infected. (H) ATPGlow showed significantly decreased cell viability after METTL3-OE and 5-FU treatment (20 μM) in AGS and MKN-45. (I) Western Blot analysis showed expression levels of PARP and Gasdermin D after treatment of METTL3-OE and 5-FU (20 μM). (J) The flow cytometry showed the apoptosis of AGS and MKN-45 after treatment of METTL3-OE and 5-FU (20 μM). (K) The gastric cancer organoid culture assay after treatment of METTL3-OE and 5-FU (20 μM). * P<0.05, ** P<0.01, *** P<0.001.

Next we performed a new RNA-seq on HP-positive GC tumor tissues with HP-negative GC tumor tissues to further explore epigenetic impact of HP on gastric cancer (3 HP+ vs 5 HP-). The m^6^A methyltransferases METTL3 and METTL14 were found to have higher fold changes in HP-positive GC tissues than HP-negative tissues, while higher expressions of YTHDF1 and YTHDF2 were also associated with HP-positive GC tissues (Fig. 2A). Cell profiling of METTL3 was performed by qRT-PCR and confirmed that its expression was highest in AGS followed by MKN-45 (Fig. 2B). The protein expressions were also consistent with the qRT-PCR results (Fig. S2A). We next validated expression level of METTL3 after HP infection and HP was found to induce METTL3 expression levels after 6h in both cell lines (Fig. 2C). WB again validated that HP infection induced expression level of METTL3 and p-P65 (Fig. 2D). So we next detect the modification level of m^6^A after HP infection. Results turned out that HP could induce m^6^A level after 6h co-culture of HP, indicating that m^6^A modification might be a self-response reaction of cells to inflammation stimulation to defend themselves (Fig. 2E). The qRT-PCR assay was performed in GC tumor tissues with or without HP infection and METTL3 had a significantly higher expression level in HP-positive tumor tissues than HP-negative (Fig. 2F). The WB analysis also showed higher expression of METTL3 in HP-infected tissues than those of non-infected tissues (Fig. 2G). The IHC staining showed similar results, suggesting higher expression of METTL3 in HP-positive GC tissues than HP-negative GC tissues (Fig. S2B).

Next, the chemotherapy sensitivity of METTL3 was investigated. We found that after overexpression of METTL3, no significant change of cell viability was demonstrated. However, cell viability in both AGS and MKN-45 cell lines was significantly reduced after 5-FU treatment and METTL3 overexpression compared to control and 5-FU treatment, suggesting that higher m^6^A modification level enhanced 5-FU sensitivity chemotherapy (Fig. 2H). Again, we detected apoptosis and pyroptosis by WB, IF, flow cytometry with treatment of 5-FU and METTL3-OE. The WB and IF analysis demonstrated that METTL3-OE could induce pyroptosis and apoptosis of AGS and MKN-45 even without 5-FU treatment, while treatment of 5-FU alone could also induce higher expression of cleaved-Gasdermin D, ASC and cleaved-PARP. When combined together, the expressions of cleaved-Gasdermin D, ASC and cleaved-PARP were significantly higher compared to other groups (Fig. 2I, Fig. S2C). The flow cytometry assay also showed similar results confirming that METTL3-OE or 5-FU treatment alone could induce higher apoptosis while combining together might induce highest apoptosis (Fig. 2J). The colony formation assay demonstrated similar results (Fig. S2D). The p-H2A.X density and ROS assay were used to detect oxidative and DNA damage of GC cells and confirmed previous results. (Fig. S2E-F). The gastric cancer organoid culture assay showed that after 5-FU treatment and overexpression of METTL3, the diameters of organoids were decreased significantly (Fig. 2K). The immunofluorescence confirmed successful model of organoid using CK7, Villin and CEA (Fig. S2G). Based on above, we found that HP infection might increase sensitivity of 5-FU by inducing expression level of METTL3.

### NK-κB regulated the transcription of METTL3

As we confirmed in the previous experiments, the expression of p-P65 was significantly increased by HP infection, indicating NF-κB might play a vital role in the regulation of METTL3 and m^6^A modification. Pathway analysis suggested NF-κB ranked 1^st^ in all activated pathways after HP infection (Fig. S3A). So we next investigated how NF-κB signaling pathway impacts expression of METTL3. The NF-κB inhibitor Bay-11-7082 and activator TNF-a were adopted based on the previous reports 38, 39. The NF-κB inhibitor Bay-11-7082 significantly depressed the expression of METTL3 while NF-κB activator TNF-a induced the expression of METTL3 (Fig. S3B). Jaspar and TFbinding bioinformatic tools were used to predict potential binding sites of p65 and promoter sequence of METTL3 (Fig. S3C). All three binding sites predicted by Jaspar were all overlapping with binding sites predicted by TFbinding (red), while TFbinding predicted 11 potential target binding sites (orange) using the 0-2000bp upstream regions of METTL3 transcription start site.

**Figure 3:**
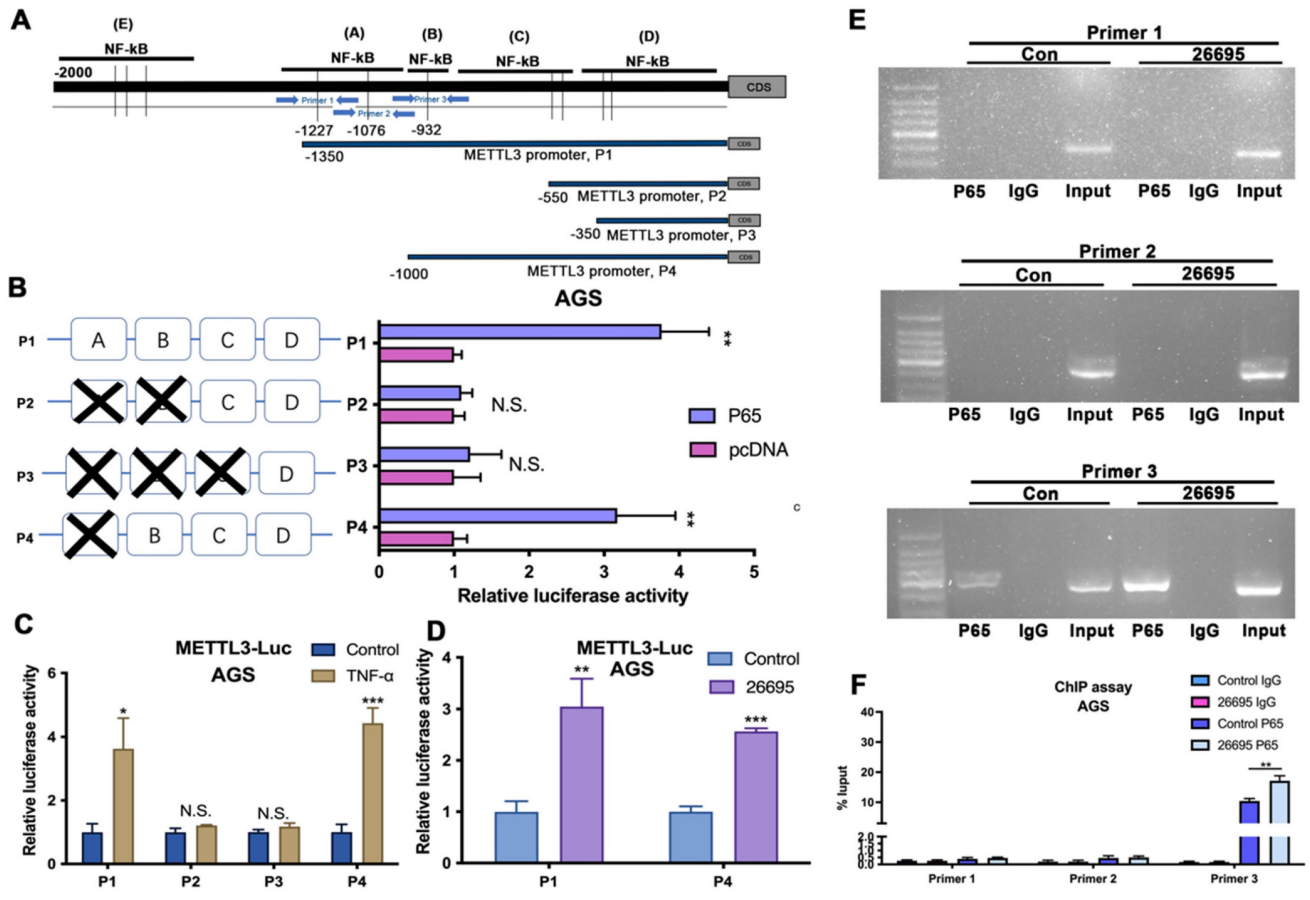
** NK-κB regulated the transcription of METTL3.** (A) Schematic diagram shows four luciferase reporters cover different DNA sequences of METTL3 promoter region for potential binding of p65. Three primers for ChIP assay are also marked with blue arrows. (B) Luciferase reporter assay analysis of four METTL3 promoter luciferase reporters in AGS cells transfected with P65 or pcDNA. (C) Luciferase reporter assay analysis of four METTL3 promoter luciferase reporters in AGS cells treated with 5 μM TNF-α (3h) before luciferase detection. (D) Luciferase reporter assay analysis of P1 and P4 in AGS cells infected with 100 MOI* H. pylori* strain 26695 for 6h. (E) RT-PCR was performed in AGS cells after pull down P65 using three pairs of primers to validate the potential P65 binding sites in METTL3 promoter region after *H. pylori* infection. (F) qRT-PCR analysis of DNA pulled down using three primers after *H. pylori* infection. * P<0.05, ** P<0.01, *** P<0.001.

According to the predictions, first we constructed three pGL3-based luciferase reporter constructs of METTL3 promoter regions containing potential binding areas (cluster A-D) named P1, P2 and P3 as shown in Figure 3A. All predicted sites were marked in the promoter sequence. After stably transfected different luciferase plasmids into AGS cells, external NF-κB-P65 plasmids and empty vector control were transfected into AGS cells for comparison and B-gal was added as base for detecting luciferase. We found that P1 which contained clusters A, B, C and D had significantly higher luciferase activity compared to control while P2 and P3 did not show significant difference compared to non-transfected AGS cells. This indicated possible binding sites were among A and B area rather than C and D. So we then constructed P4 luciferase plasmid which contained binding sites located in B, C, and D area. Luciferase results showed that P4 also demonstrated a higher density compared to non-transfected cells. Combining with previous results, this suggested that the area B could be the precise binding site (Fig. 3B). To further validate, we used external TNF-a instead of internal P65 to activate luciferase signals (Fig. 3C). Consistent with previous results, P1 and P4 showed significantly higher luciferase density than vector while no differences were observed in P2 and P3. Next, the 26695 strain was used for further treatment on AGS cells transfected with P1 and P4 and positive luciferase density was observed (Fig. 3D). These results confirmed that HP would enhance the binding of METTL3 promoter and NF-κB-P65 with the validated binding site B, therefore leading to higher expression of METTL3. These results strongly suggest the functional NF-κB binding sites on METTL3 promoter, localized in cluster B of METTL3-P1 and METTL-P4, that play an active role in gastric cells.

To further probe the direct NF-κB-p65 binding site to the METTL3 promoter region in AGS, we designed three pairs of primers for amplification to detect the binding using chromatin immunoprecipitation (ChIP) assay (Fig. 3A). After HP infection, the RT-PCR demonstrated that Primer 3 could blast products, validating the -840bp of METTL3 promoter was the binding site of NF-κB (Fig. 3E-F).

### HP-induced METTL3 regulates 5-FU sensitivity via its downstream target BRD2

**Figure 4:**
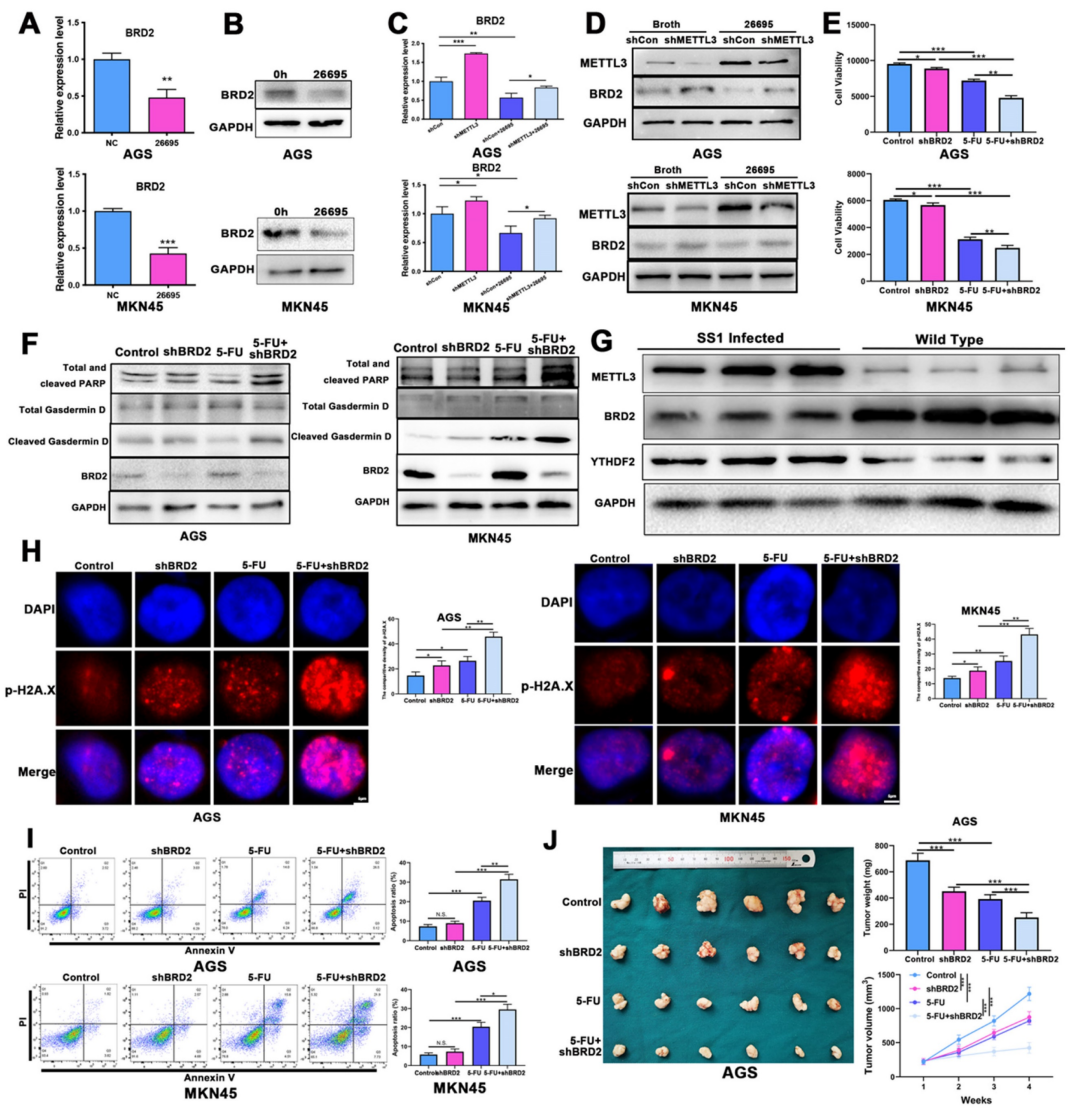
** HP-induced METTL3 regulates 5-FU sensitivity via its downstream target BRD2.** (A) The qRT-PCR showed decreased expression level of BRD2 after *H. pylori* infection for 6h in AGS and MKN-45. (B) The WB analysis showed decreased expression level of BRD2 after *H. pylori* infection for 6h in AGS and MKN-45. (C) The qRT-PCR expression level of BRD2 after knockdown of METTL3 and *H. pylori* infection in AGS and MKN-45. (D) The WB analysis of BRD2 after knockdown of METTL3 and *H. pylori* infection in AGS and MKN-45. (E) The ATPGlow assay showed cell viability after knockdown of BRD2 and *H. pylori* infection in AGS and MKN-45. (F) The WB analysis of PARP, Gasdermin D and BRD2 after knockdown of BRD2 and treatment of 5-FU. (G) WB showed expression levels of METTL3, YTHDF2 and BRD2 in Tff1-KO mice treated with and without SS1 strain. (H) The IF showed p-H2A.X staining of AGS and MKN-45 after knockdown of BRD2 and treatment of 5-FU. (I) The flow cytometry analysis of apoptosis of AGS and MKN-45 after knockdown of BRD2 and treatment of 5-FU. (J) The nude mice model of tumor analysis after knockdown of BRD2 and treatment of 5-FU. * P<0.05, ** P<0.01, *** P<0.001.

We then focused the downstream target of METTL3 in the role of regulating 5-FU sensitivity. We constructed knockdown and overexpression model of METTL3 in GC cells and the efficacy was verified by qRT-PCR and WB (Fig.S3D-E). We performed m^6^A-seq with HP-positive tumor tissues vs HP-negative tumor tissues (3 vs 3) to detect variations caused by HP and 1178 targets were retrieved (Fig. S4A-B). The online bioinformatics tool was also adopted and we found that number of possible targets that were methylated by METTL3 was 2358 in total (Fig. S4C). We again re-analyzed the NGS data to find possible differently-expressed genes caused by HP infection and 474 targets were found (Fig. S4D). After overlapping these 3 results, only PPP1R15A, BRD2 and ZNF182 emerged as m^6^A-methylated targets by HP, as well as the predicted targets of METTL3 and upregulated by HP (Fig. S4A). After initial filtering, we confirmed BRD2 as the downstream target of HP-induced METTL3 as results confirmed no expression change after HP infection for other targets (Fig. S4E). Next, we validated whether HP infection could affect expressions of BRD2. Western blot and qRT-PCR confirmed that 26695 infection could significantly decrease the expression of BRD2 in AGS and MKN-45 cell lines (Fig. 4A-B). After knockdown of METTL3, the expression of BRD2 was increased both in mRNA and protein level in AGS and MKN-45 cell lines. When co-culture with 26695, expression of BRD2 was significantly lower than control. After combining treatments of knockdown of METTL3 and 26695 strain, the expression of BRD2 was stronger than control but weaker than knockdown of METTL3 treated alone. We used another shMETTL3 and the WB results turned out to be the same (Fig. 4C-D, Fig. S4F).

Next we used MeRIP-PCR to detect the m^6^A modification level of BRD2 to input and knockdown of METTL3 significantly decrease the m^6^A modification level of BRD2, which was consistent with our previous results. These outcomes above validated that BRD2 was regulated by METTL3 and was possibly the key downstream target to regulate cell death process (Fig. S4G).

The expression of BRD2 was then validated in 30 human GC tissues and 30 human normal tissues and it was significantly higher than tumor than in normal tissues (Fig. S4H). BRD2 was then validated in 17 HP-positive and 17 HP-negative GC tumor tissues and BRD2 was highly expressed in HP-negative GC tissues (Fig. S4I). The IHC analysis also showed that BRD2 was highly expressed in HP-negative tissues than HP-positive GC tissues (Fig. S4J).

Next, to assess the effects on cell deaths of BRD2, we conducted *in vitro* and *in vivo* experiments to validate. After combination of 5-FU and knockdown of BRD2, the ATPglow assay in AGS and MKN-45 showed that cell viability was significantly dropped to lowest level compared to other groups, indicating knockdown of BRD2 sensitized GC cells to 5-FU (Fig. 4E). Moreover, WB and IF confirmed that knockdown of BRD2, expressions of cleaved PARP, ASC and Cleaved-Gasdermin D both increased, and after treated with knockdown of BRD2 and 5-FU, apoptosis and pyroptosis were induced to the highest levels (Fig. 4F, Fig. S4K). These results suggested that BRD2 might play an important role in regulating apoptosis and pyroptosis.

We then used TFF1-KO mice for *in vivo* analysis of BRD2 and METTL3 (Fig. 4G). We first applied SS1 strain for oral administration into TFF1-KO mice for HP infection. After 1 month of infection, mice were sacrificed and protein was extracted. WB showed that METTL3 was highly expressed in SS1 infected mice, which was consistent with previous results. BRD2 was highly expressed in WT mice, which was negatively related with METTL3. The qRT-PCR assay on SS1-treated mice showed the HP could induce the expression level of METTL3 and down-regulated BRD2 expression (Fig. S4L).

Organoid culture assay of gastric cancer cells also demonstrated that after knockdown of BRD2, the growth of GC organoid was inhibited significantly compared to 5-FU treated alone or knockdown of BRD2 alone, confirming sensitivity of 5-FU changing *in vitro*. After combination of 5-FU and knockdown of BRD2, the growth of organoid was most inhibited compared to other groups (Fig. S4M). The ROS assay and immunofluorescence were applied to validate the oxidative stress and DNA damage again. Similar results were obtained using the ROS and p-H2A.X staining, suggesting that knockdown of METTL3-regulated BRD2 could enhance the oxidative stress and DNA damage after 5-FU treatment (Fig. 4H, Fig. S4N]). Flow cytometry also confirmed that after knockdown of BRD2, apoptosis in 5-FU treated AGS and MKN-45 cells was upregulated most compared to other groups (Fig. 4I). The colony formation assays also confirmed that 5-FU and shBRD2 treatment induced strong cell deaths (Fig. S4U). In the nude mice model, AGS cells transfected with shBRD2, 5-FU and a combination of shBRD2 and 5-FU were injected into nude mice separately. After 6 weeks of observation, mice injected with shBRD2 demonstrated smaller tumor than control, but bigger than tumor treated with 5-FU alone (Fig. 4J).

We also performed rescue experiments to validate verify the upstream and downstream relationships between them. After HP infection, the apoptosis and colony formation were induced in AGS and MKN-45 after 5-FU treatment but knockdown of METTL3 could inhibit the apoptosis (Fig. S5A-B). Likewise, the overexpression of METTL3 could induce apoptosis and colony formation while overexpression of BRD2 reversed the effects, suggesting METTL3 could regulate apoptosis via expression levels of BRD2(Fig. S5C-D).

### METTL3 regulated BRD2 m^6^A methylation via YTHDF2-dependent manner

Interestingly, in the NGS analysis, we found expressions of m^6^A-specific binding proteins YTHDF1 and YTHDF2 were also significantly increased by HP infection, which triggered us to investigate if YTHDF family proteins play important roles in BRD2 m^6^A modification process. The qPCR assay confirmed expression levels of both YTHDF1 and YTHDF2 were upregulated after 3h and 6h of HP infection (Fig. S6A). We next used RMBase to predict possible recognizer protein of BRD2 and results turned out that both YTHDF1 and YTHDF2 could bind to BRD2 (Fig. S6B). The TCGA data suggested that low expression of YTHDF1 and high expression of YTHDF2 were associated with better prognosis (Fig. S6C). We knocked down YTHDF1 and YTHDF2 and expression of BRD2 was observed to be significantly increased both in mRNA and protein level after YTHDF2 knockdown (Fig. S6D-E). This indicated that YTHDF2 may also participate in the BRD2 modification. We also explored of the role of 26695 and YTHDF2 in modulating half-life of BRD2 mRNA and the results showed that 26695 promoted BRD2 degradation, and this effect could be rescued by knocking down YTHDF2 (Fig. S6F). Next, we further examined the capacity of YTHDF2 binding to m^6^A modified BRD2 mRNA and we found that when METTL3 was knocked down, the capacity of YTHDF2 binding to BRD2 decreased (Fig. S6G).

**Figure 5:**
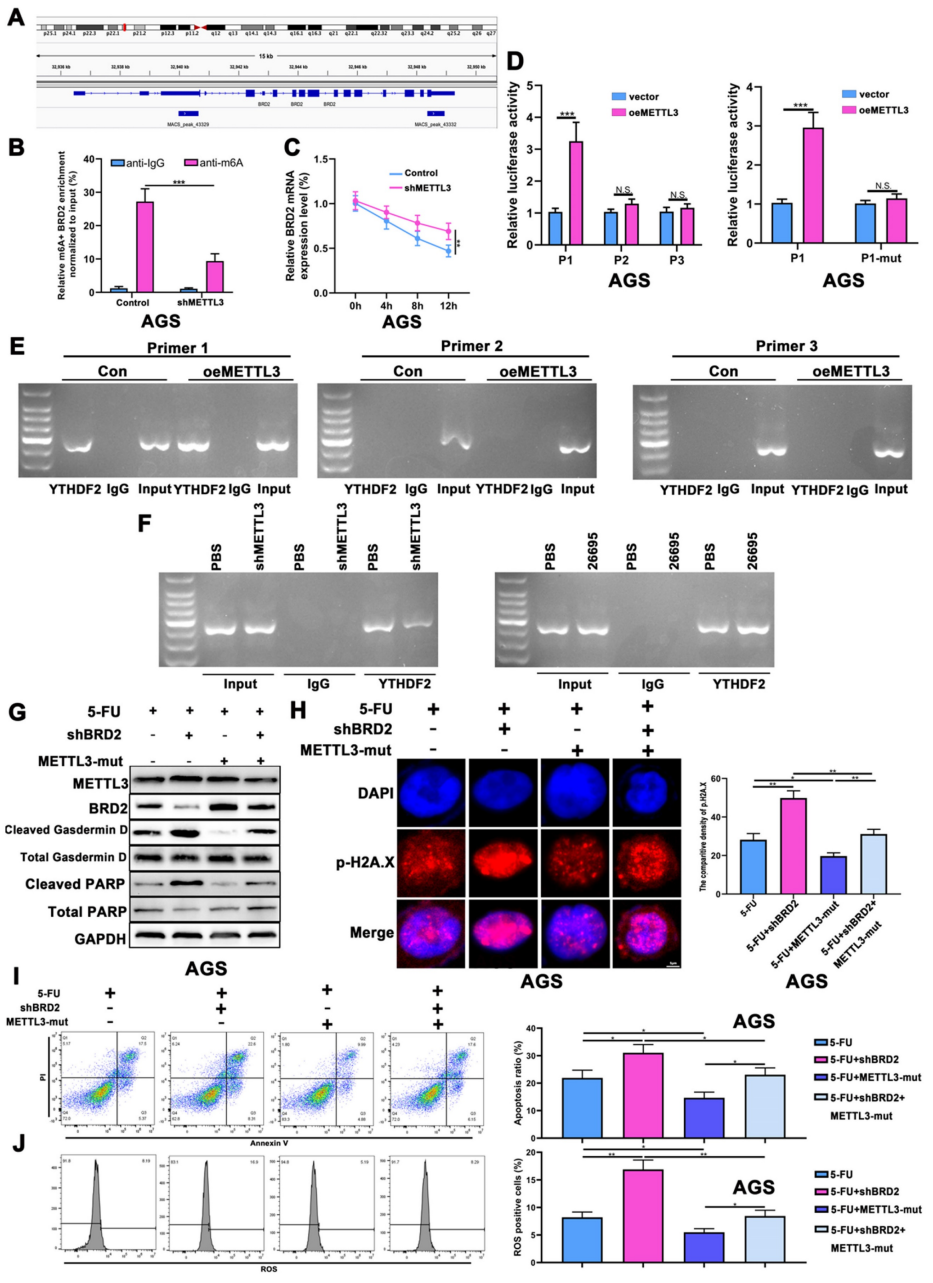
** METTL3 regulated BRD2 m^6^A methylation via YTHDF2-dependent manner.** (A) The m^6^A-seq data presented by IGV tool. (B) The relative m^6^A methylation level of BRD2 after knockdown of METTL3. (C) The actinomycin D experiment suggested that knocking down METTL3 increased the stability of BRD2. (D) Luciferase assay showed luciferase activities of P1, P2 and P3 after overexpression of METTL3. After transfected with P1-mut plasmids, the luciferase activity decreased compared to P1 after overexpression of METTL3. (E) RT-PCR was performed in AGS cells after pull down YTHDF2 using three pairs of Primers targeting P1, P2 and P3. (F) RT-PCR was performed in AGS cells after pull down YTHDF2 using Primer 1 after knockdown of METTL3 and *H. pylori* infection. (G) WB analysis showed expression levels of METTL3, BRD2, PARP and Gasdermin D after 5-FU treatment, knockdown of BRD2 and METTL3-mut. (H) The IF staining of p-H2A.X showed densities of METTL3, BRD2, PARP and Gasdermin D after 5-FU treatment, knockdown of BRD2 and METTL3-mut. (I) The flow cytometry of apoptosis analysis of METTL3, BRD2, PARP and Gasdermin D after 5-FU treatment, knockdown of BRD2 and METTL3-mut. (J) The flow cytometry of ROS analysis of METTL3, BRD2, PARP and Gasdermin D after 5-FU treatment, knockdown of BRD2 and METTL3-mut. *, P<0.05, **, P<0.01, ***, P<0.001.

Based on the m^6^A-seq data from IGV tool, two binding sites are expected to be located in CDS region and 3'UTR regions, of which BRD2 binding sites have not been identified (Fig. 5A, Fig. S6H). According to the prediction sites of RMBase for BRD2 and YTHDF2, we noticed all 3 predicted sites were located on the CDS region (2) and 3'UTR region (1), which were marked in colors. We used RIP assay to detect the binding of different sites of YTHDF2. The MeRIP-PCR was adopted to validate that after knockdown of METTL3, the relative m^6^A methylation level was decreased compared to control (Fig. 5B). The actinomycin D experiment suggested that knocking down METTL3 increased the stability of BRD2 mRNA (Fig. 5C). Three luciferase reporter constructs containing different sequence of predicted binding sites were transfected into AGS cells, named as P1, P2 and P3, respectively. After overexpression of METTL3, luciferase assay showed P1 had a significantly higher activity than empty vector. To be noted, we did not detect increased luciferase activity of P2 and P3, indicating P1 could be the potential binding site of YTHDF2 and BRD2(Fig. 5D). Next we mutated the P1 sequence and validated the luciferase activity after overexpression of METTL3. The outcomes turned out that P1-mut would not result in significant difference in luciferase (Fig. 5D). We then detected the binding using chromatin immunoprecipitation (ChIP) assay. The DNA pulldown by beads containing YTHDF2 antibody in AGS cells was utilized as a template in the RT-PCR reaction. The PCR results revealed amplification of the pull downed DNA region using Primer 1 was significantly higher than P2 or P3 after METTL3-OE, no PCR products emerged on P2 and P3 but those of P1 showed that BRD2 could bind with YTHDF2 through P1 and METTL3 could enhance the binding (Fig. 5E). We then knocked down METTL3 using Primer 1 following the same conditions. The PCR expression was lower after knockdown of METTL3 compared to those treated with PBS(Fig. 5F). Next, we investigated if HP infection would affect the binding of YTHDF2 and BRD2. After 26695 infection, ChIP was performed again using YTHDF2 antibody and the PCR results showed higher expression in HP infection group than PBS group (Fig. 5F).

After validating the binding of YTHDF2 and BRD2, we detected the apoptosis and pyroptosis regulated by METTL3-BRD2 axis. The knockdown of BRD2 would induce cleaved Gasdermin D and cleaved PARP after 5-FU. To further validate whether the m^6^A methyltransferase activity of METTL3 regulates gastric carcinogenesis via BRD2, we introduced two key point mutations (D395A and W398A) into METTL3 to abolish its catalytic capacity (Fig. S6I). After mutation of METTL3, BRD2 was upregulated, leading to lower expression of cleaved Gasdermin D and cleaved PARP. When combining knockdown of BRD2 and METTL3-mut, we found that expression of BRD2 was not significantly changed compared to control, as well as Gasdermin D and PARP after 5-FU treatment (Fig. 5G, Fig. S6J). The IF staining of p-H2A.X after 5-FU treatment also showed that DNA damage was increased after shBRD2 and decreased after METTL3-mut. While combining shBRD2 and METTL3-mut, the DNA damage was lower than shBRD2 treated alone but higher than METTL3-mut treated alone, which was consistent with previous results (Fig. 5H). The ROS assay and flow cytometry were used to detect the METTL3-BRD2-regulated DNA damage and apoptosis. After applying 5-FU, DNA damage and apoptosis were both induced after knockdown of BRD2 and suppressed after transfected with METT3-mut. Again, when combining shBRD2 and METTL3-mut, the apoptosis and DNA damage were not significantly changed compared to control as well (Fig. 5I-J).

### Knockdown of BRD2 activated Caspase-8 via FOXO4-FLIP axis

**Figure 6:**
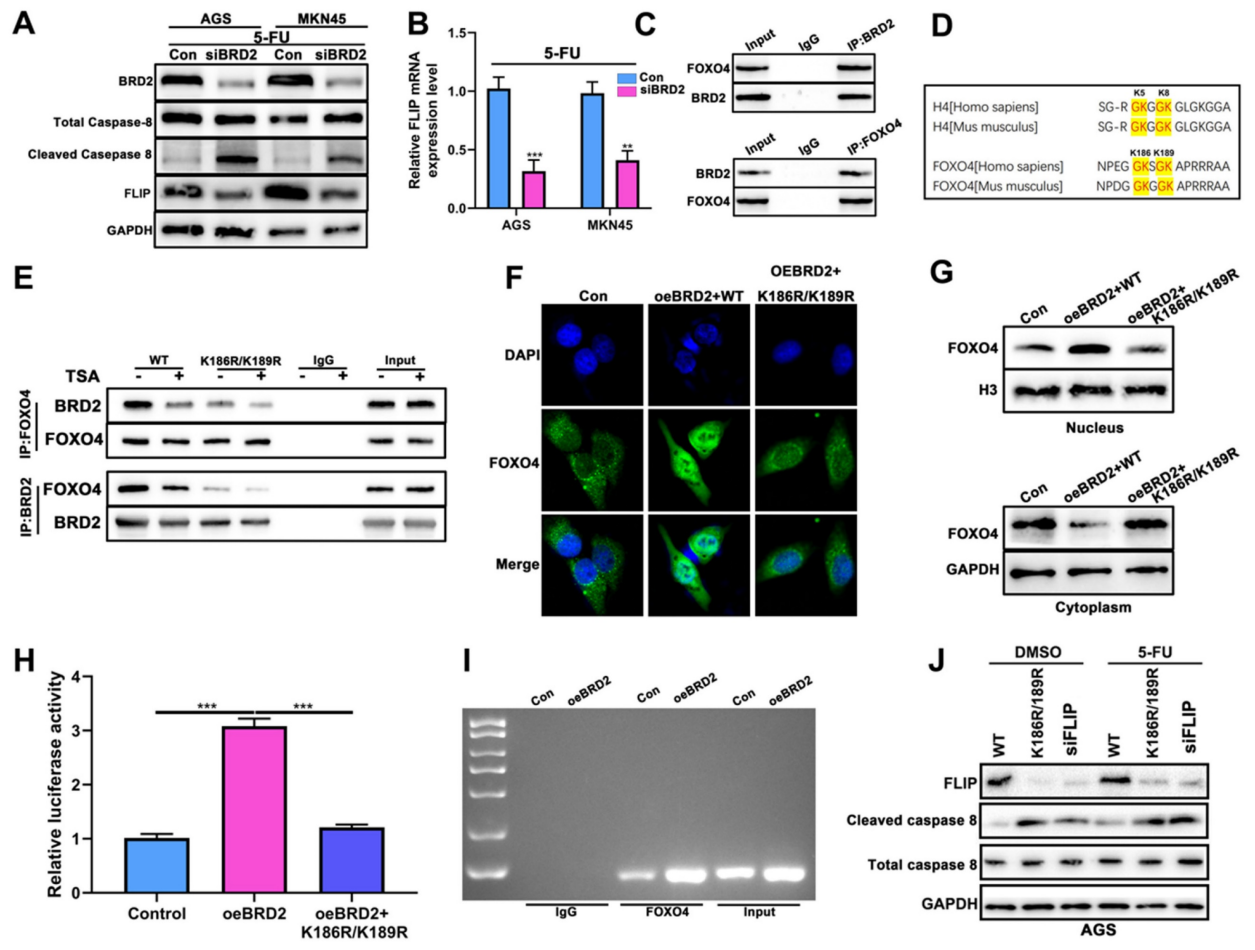
** Knockdown of BRD2 activated Caspase-8 via FOXO4-FLIP axis.** (A) The WB analysis of Caspase-8 and FLIP after knockdown of BRD2 and 5-FU treatment in AGS and MKN-45. (B) The mRNA analysis of FLIP after knockdown of BRD2 and 5-FU treatment in AGS and MKN-45. (C) The IP analysis of BRD2 and FOXO4. (D) Schematic diagram showing the highly conserved GK-X-GK motif in K5/8 of histone H4 and K186/K189 of FOXO4. (E) The IP analysis of BRD2 and FOXO4 after transfection of K186R/K189R. (F) The IF analysis of FOXO4 after overexpression of BRD2 and ransfection of K186R/K189R. (G) The nucleus and cytoplasm WB analysis of FOXO4. (H) The Luciferase reporter assay after overexpression of BRD2 and transfection of K186R/K189R. (I) The qPCR results of pull down FOXO4 to validate the potential FOXO4 binding sites in FLIP promoter region after overexpression of BRD2. (J) The WB analysis of FLIP and Caspase-8 after treatment of knockdown of BRD2 K186R/K189R. *, P<0.05, **, P<0.01, ***, P<0.001.

As shown before, pyroptosis and apoptosis were both induced after HP infection. In 2019, it was reported that apoptosis-inducing protein Caspase-8 might also induce pyroptosis 30. Therefore, we proposed that Caspase-8 could be the switch of both apoptosis and pyroptosis. WB analysis showed that after knockdown of BRD2, cleaved Caspase-8 was highly induced after treatment of 5-FU in both AGS and MKN-45 (Fig. 6A). After filtering NGS data comparing HP+ tumor vs HP- tumor, FLIP emerged into our sight with decreased expression level after HP infection. Reports showed FLIP could compete with Caspase-8 to maintain the homeostasis of cell death. We propose that this might be the key of switching apoptosis and pyroptosis. After knockdown of BRD2, the expression of FLIP increased as well as cleaved Caspase-8 (Fig. 6A), which was also validated by qRT-PCR test (Fig. 6B). Based on above, FLIP could possibly become the key factor in regulating Caspase-8.

We then verified the potential interactions of BRD2 and FLIP. Considering BRD family is capable of binding Histone H4 without being the transcription factor 40, we first performed the COIP-MS of BRD2 and overlap the results with the potential transcription factors of FLIP for further interacting targets (Fig. S7A-B). The overlapping analysis showed FOXO4 was the potential transcription factor. The IP analysis showed strong binding of BRD2 and FOXO4 (Fig. 6C).

It has been previously established that lysine residues at positions 186 and 189 of FOXO4, situated proximal to the nuclear localization signal (NLS) of the FOXO protein family, represent a histone H4 mimic GK-X-GK motif (Fig. 6D) 41, 42. Given the pivotal interaction of acetyl lysine residues on histone H4 K5/K8 and their affinity for the BD1 domain of the BET protein family, it is hypothesized that the di-acetylation at K186/K189 of FOXO4 is critical for its binding to BRD2. We constructed both the wild-type (WT) and mutant FOXO4(K186R/K189R), wherein the lysine residues at positions 186 and 189 in the mutant variant were substituted with arginine. Subsequently, the binding affinity between BRD2 and FOXO4 was assessed using co-immunoprecipitation assays. Within our expectation, the binding capacity of BRD2 and mutant FOXO4 variant was decreased in both BRD2 and FOXO4 IP (Fig. 6E).

The mechanism was further studied. The nucleus staining of FOXO4 showed overexpression of BRD2 enhanced the nuclear import of FOXO4 and reversed after transfected with K186R/K189R (Fig. 6F). Next we studied the FOXO4 expression in the nucleus and cytoplasm after overexpression of BRD2 and K186R/K189R mutation. WB showed BRD2 could increase the expression of FOXO4 in the nucleus and decease in cytoplasm. After transfected with K186R/K189R, the import to nucleus of FOXO4 was blocked and remained in the cytoplasm (Fig. 6G). As shown in Fig. S7C, the motif was predicted with highest scores at -1669bp as the binding site of FLIP promoter and FOXO4. The overexpression of BRD2 increased the luciferase activity after transfected luciferase plasmids containing the potential binding sites. After transfected with K186R/K189R, the luciferase decreased compared to overexpression of BRD2 alone (Fig. 6H). The ChIP assay also indicated that overexpression of BRD2 could enhance the binding of FLIP and FOXO4 (Fig. 6I), therefore regulating Caspase-8 switch. The downstream Casapse-8 would be regulated by the expression of BRD2/FOXO4/FLIP axis. WB showed that after knockdown of BRD2 or transfected with K186R/K189R, the expression of FLIP both decreased significantly. Meanwhile, cleaved-Caspase 8 were significantly increased compared to control, indicating that FLIP might compete with Caspase-8 for the binding of FOXO4. After 5-FU treatment, we noticed that expression of cleaved-Caspase 8 was significantly higher than DMSO-treated group and expression of FLIP still remained the same (Fig. 6J). These results indicated BRD2/FOXO4/FLIP/Caspase-8 axis could be the mechanism of BRD2 regulating apoptosis and pyroptosis.

## Discussion

*H. pylori* is well known as the primary carcinogen of gastric cancer 6, 43. Statistics indicates that half the population on earth are infected with HP, owing to oral and food transmission 9, 43. Eradication of HP should be applied for non-gastric cancer people as a prevention of gastric cancer 5, 6, 43. Nevertheless, certain studies have indicated that administering chemotherapy to gastric cancer patients with a positive HP infection may not be detrimental and could potentially enhance the sensitivity of 5-FU in gastric cancer chemotherapy 11, 12. In the current study, we employed 5-FU to assess the viability of GC cell lines which remains a standard therapeutic approach in treatment of gastric cancer chemotherapy. Since the underlying mechanism of 5-FU involves targeting the DNA double strand structure whereas BRD2 exhibited a robust ability to repair the double strand damage, we adopted 5-FU in this study. In the present study, we found that cell viability was significantly lower with HP infection after 5-FU treatment, indicating a better sensitivity of 5-FU of HP-positive GC cells. This suggested that HP infection might play a different role in normal people and gastric cancer patients, which could affect the possible treatment plan for HP+ gastric cancer patients. To be noted, we did the OS analysis on advanced gastric cancer patients undergoing chemotherapy including 5-FU with and without HP infection but no significance was demonstrated. This might result from the relatively small sample size and mixed chemotherapy used in patients rather than 5-FU alone.

In the previous report, Cheng *et al.* reported that HP infection was associated with the process of FTO-mediated m^6^A modification promoting malignant transformation of gastric mucosal epithelial cells, but the role remains unclear 44. In the present study, we validated that HP could induce m^6^A methylation in gastric cancer cell lines, which is the first report to reveal the mechanism in gastric cancer to our best knowledge. Even though in Cheng's study, m^6^A methylation was involved in the malignant transformation after HP infection while our study mainly focuses on the role of HP infection on gastric cancer cell lines, we still could assume that HP was able to induce m^6^A methylation. This might be part of the process of self-response of gastric cells after HP infection, therefore regulating resistance to chemotherapy. Differently, several reports confirmed METTL3 could be the major reason of chemotherapy sensitivity regulation, rather than FTO, which was also validated by our results 23, 24. BRD2 is confirmed as a vital protein in regulating apoptosis in gastric cancer reported in our previous study 33. We extended its underlying functions from apoptosis to another cell death process pyroptosis via various experiments. The findings suggested that BRD2 might be the key to chemotherapy resistance, which was beyond our initial expectations. We also first discovered that METTL3 and YTHDF2 could coordinately regulate m^6^A modification of BRD2 and determine the potential binding sites of BRD2 and YTHDF2. This might not only be of helpful for the BRD2 regulation during HP infection process but also extended the interaction network of METTL3-regulated chemotherapy sensitivity.

Moreover, in the present study, BRD2 regulated Caspase-8 via FLIP and the further mechanisms were discovered in how BRD2-regulated Caspase-8 induce apoptosis and pyroptosis, which were reported by various studies 28, 31. Studies showed that Caspase-8 was the molecular switch of inducing pyroptosis, apoptosis and necrosis, also called PANoptosis 30. This is also the first report to show that BRD2 could regulate Caspase-8 to induce PANoptosis in gastric cancer. Previous reports have confirmed FLIP had similar functions as Caspase-8, and it was considered as pseudo-caspase 37. As a result, FLIP could compete with Caspase-8 and it was also highly involved in the regulation of apoptosis and pyroptosis in GC cell lines. Therefore, we considered that FLIP could be the downstream target of BRD2 regulatory axis. As the bioinformatics prediction of FLIP was overlapping with the proteomics analysis of FLIP via CO-IP, FOXO4 came into our sight. Previous reports had already confirmed that BET family could recognize H4-K5ac/K8ac peptide and had a very strong affinity to accylation loci 40, 45. Interestingly, another report emerged about the similar structure of FOXO4 and histone H4-K5ac/K8ac, which could be the cause that FOXO4 could strongly bind to BRD2 in our results. It is reported that acetylation is also involved in the FOXO4 transcriptional activity and K186, K189 are the acetylation sites similar to Histone H4 K5ac/K8ac. Based on the prediction, we then mutated the acetylation sites at K186 and K189 transfected cells with these constructs. We found the binding of BRD2 and FLIP-promoter FOXO4 was inhibited resulting in the decreased expression of FLIP, while cleaved Casepase-8 was activated. This piece of evidence suggested that transcription of FLIP was blocked by the mutates of the transcription factor FOXO4. This is also the first time to confirm that BRD2 could inhibit FLIP by blocking the binding to the FLIP-promoter FOXO4, therefore activating the Capase-8 switch to induce apoptosis and pyroptosis.

Certainly, our study has some limitations. Even though we have validated in cells that HP-positive cells demonstrated better chemotherapy sensitivity, it is not solid to draw the same conclusion in patients as the survival data did not show significant difference as standard chemotherapy is more complicated. Although we reported the mechanism of BRD2 regulating PANoptosis, more underlying mechanisms could be further discovered as BRD2 could bind to multiple TFs and transcriptional regulations might not be only limited to FOXO4-FLIP pathway.

At present, BET family proteins have become one of the new targets for scientists to develop innovative cancer therapies. It is the first time to report that BRD2 could promote the nucleus import of FOXO4, which again extended the functions of BRD2, making BRD2 again the potential therapeutic target of future gastric cancer chemotherapy. In our study, we identified BRD2 as a key regulator of chemotherapy in HP-positive gastric cancer. There may be more studies about the BET family in future cancer research.

## Conclusion

Our study confirmed the HP-induced m^6^A methylation could sensitized gastric cancer to 5-FU with activation of caspase-8 and induced apoptosis and pyroptosis. NGS and m^6^A-seq confirmed that BRD2 activated by NF-κB pathway was the target of methylation and regulates Capase-8 by binding to FLIP-promoter FOXO4. This study provides new sights to the HP-positive gastric cancer chemotherapy.

## Supplementary Material

Supplementary figures and tables.

## Abbreviations

BRD2: Bromodomain-containing protein 2
METTL3: Methyltransferase 3
YTHDF2: YTH N6-methyladenosine RNA binding protein F2
ROS: Reactive oxygen species
H. pylori: Helicobacter pylori
WB: Western blots
qRT-PCR: Quantitative real-time PCR
ChIP: Chromatin Immunoprecipitation
5-FU: 5-Fluorouracil
GC: Gastric cancer
Redox: Oxidation and reduction
FBS: fetal bovine serum
DAPI: 4′, 6-diamidino-2-phenylindole
IHC: Immunohistochemistry
IF: Immunofluorescence
TCGA: The Cancer Genome Atlas
ASC: Apoptosis-associated speck-like protein
