# Supplementary material for: Helicobacter Pylori-induced BRD2 m6A modification sensitizes gastric cancer cells to chemotherapy by breaking FLIP/Caspase-8 homeostasis
Source: Int J Biol Sci. 2025 Jan 1;21(1):346–62. doi: 10.7150/ijbs.97464 (PMC11667809; doi:10.7150/ijbs.97464)
Supplement: Supplementary file 1 — Supplementary figures and tables. [file ijbsv21p0346s1.pdf]

Figure S1

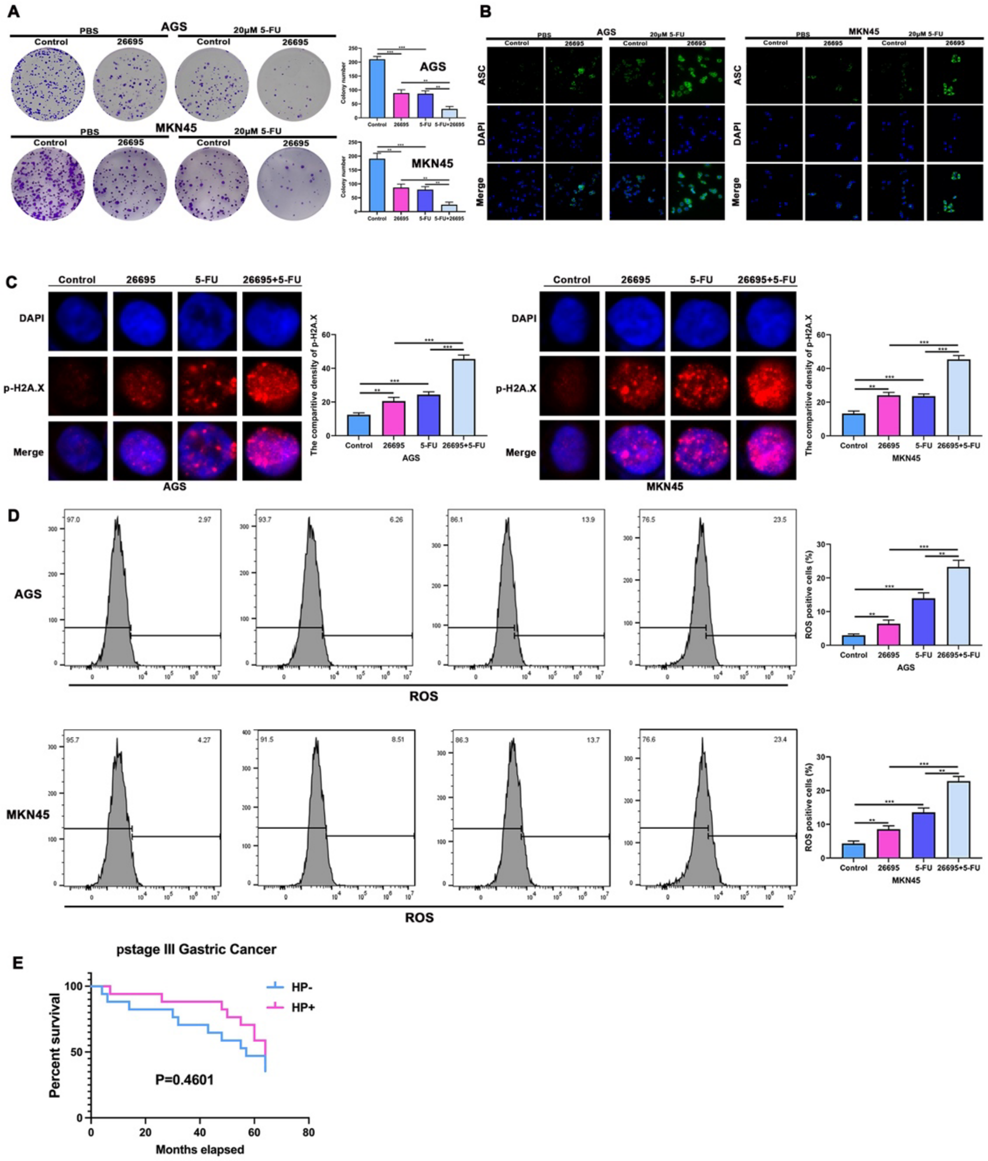

HP infection sensitized GC cell lines AGS and MKN-45 to 5-FU treatment and induced higher DNA damage and oxidative stress

(A) The colony formation of HP-infected or uninfected GC cell lines after treated with PBS or 20uM 5-FU. (B) Immunofluorescence analysis of ASC in HP-infected or uninfected GC cell lines treated with PBS or 20 μM 5-FU. (C) The level of p-H2A.X was detected using 5-FU treatment with or without *H. pylori* infection after 6h in AGS and MKN-45 cell lines. (D) The ROS assay showed different ROS density after treatment of HP infection and 5-FU in AGS and MKN-45 cell lines. (E) Kaplan-Meier analysis of overall survival (PFS) in p-stage III gastric cancer patients who received 5-FU chemotherapy (17 vs 17) based on HP infection status. * P<0.05, ** P<0.01, *** P<0.001.

Figure S2

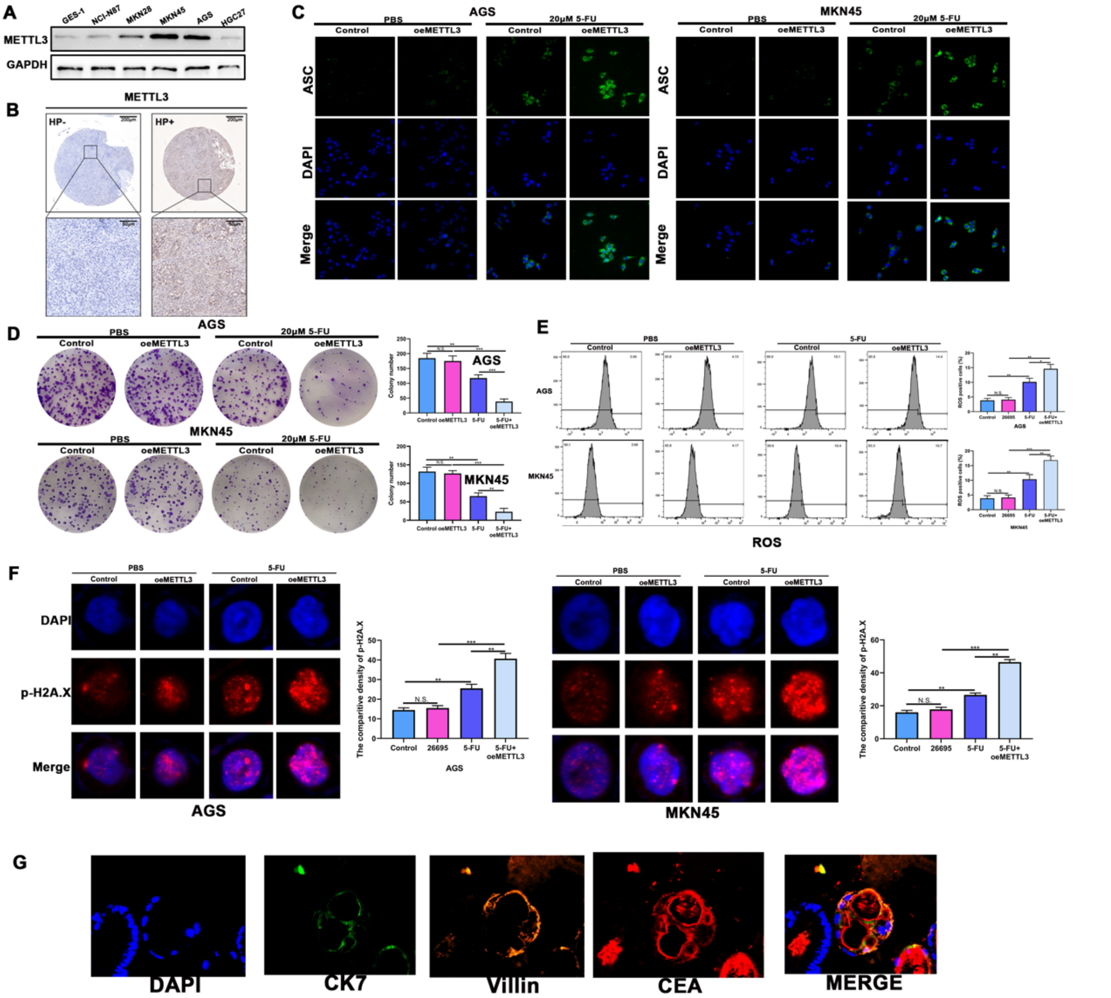

The overexpression of METTL3 sensitized GC cells to 5-FU treatment and induced more DNA damage

(A) The cell profiling of METTL3 by Western Blot. (B)The immunohistochemistry analysis showed expressions of METTL3 in gastric cancerous tissues with or without HP infection. (C) The immunofluorescence staining demonstrated different p-H2A.X expressions after treatment of 5-FU(20 μM) and METTL3-OE. (D) The colony formation assay of AGS and MKN-45 after 5-FU and METTL3-OE treatment. (E). The ROS assay showed different ROS density after treatment of overexpression and 5-FU. (F) The IF of p-H2A.X showed DNA damage after treatment of overexpression and 5-FU. (G) The IF staining(CK7, Villin and CEA) of gastric cancer organoid culture .* P<0.05, ** P<0.01, *** P<0.001.

Figure S3

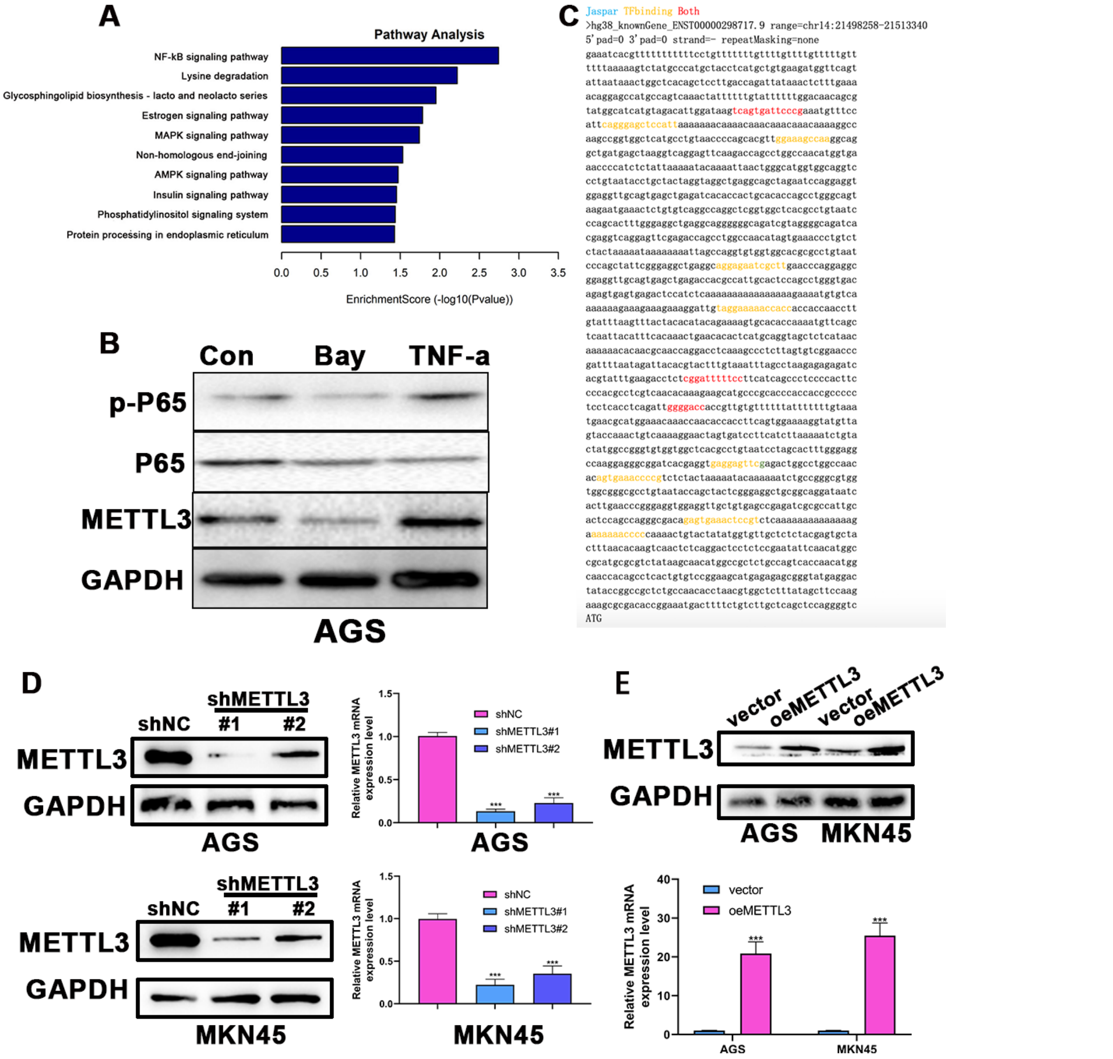

The prediction of binding sites between p-P65 and METTL3 promoter and validation of shMETTL3

(A) The NGS-seq pathway analysis showed NF-κB signaling pathway ranked top between HP-positive/HP-negative gastric cancer tissues. (B) WB showed p-P65 and METTL3 expression after treatment of 5 μM TNF-α (3h), Bay-11-7082(6h). (C) The potential binding sites of NF-κB on promoter sequence of METTL3 predicted by Jaspar(blue) and TFbinding(yellow). Red indicated binding sites predicted by both. (D) The WB and qRT-PCR analysis confirmed both shMETTL3 effective in AGS and MKN-45 cell lines. (E) The WB and qRT-PCR analysis confirmed oeMETTL3 effective in AGS and MKN-45 cell lines. * P<0.05, ** P<0.01, *** P<0.001.

Figure S4

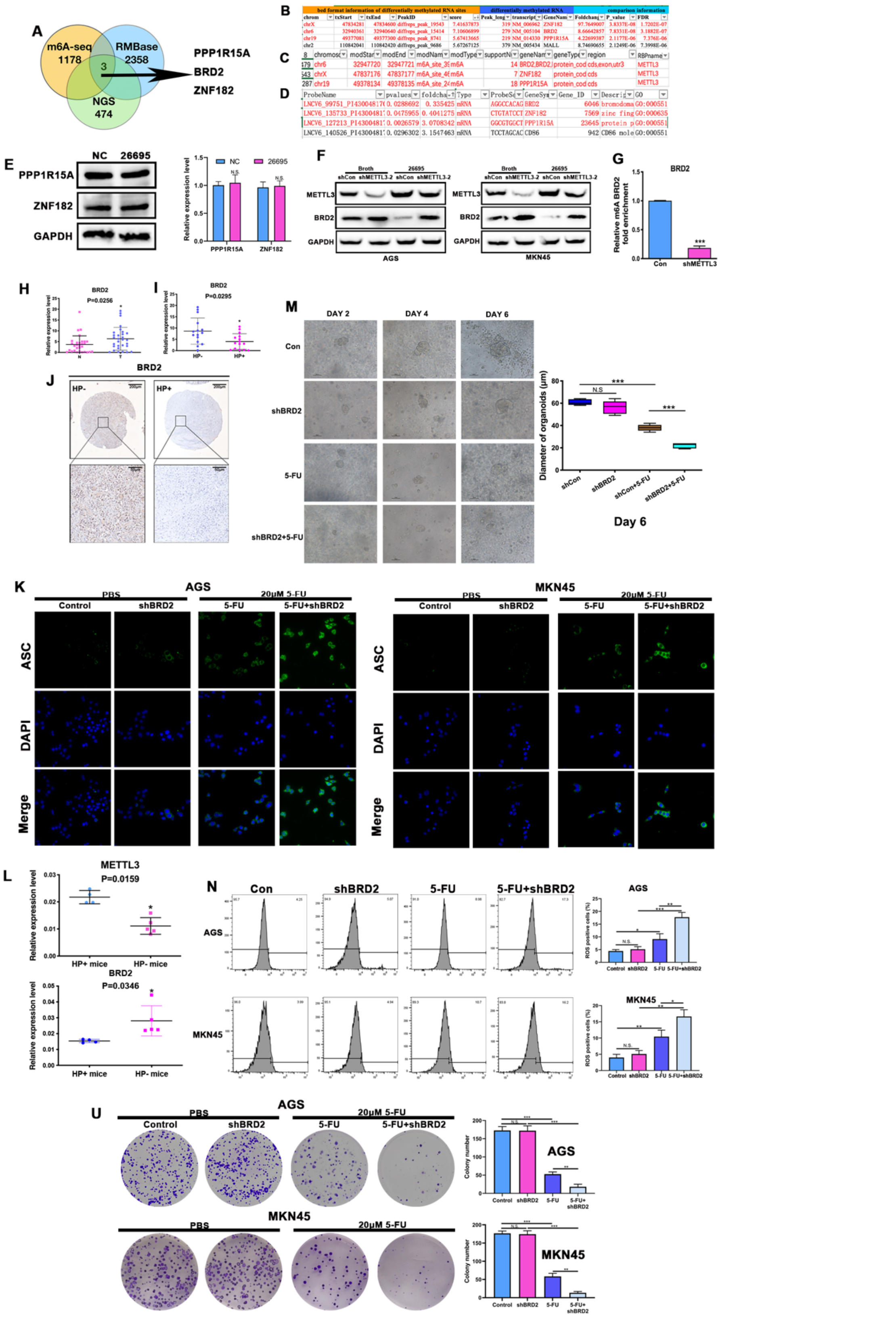

The prediction of the downstream target BRD2 and the knockdown of BRD2 sensitized GC cells to 5-FU treatment

(A) Three overlapping potential targets were predicted by NGS-seq, RMBase and m^{6}A

seq. (B) Analysis of m^{6}A-seq. (C) Analysis of RMBase prediction. (D) Analysis of NGS-seq. (E) The WB and qRT-PCR analysis showed negative results of other potential targets. (F) The WB analysis showed BRD2 was regulated by METTL3 after HP infection and shMETTL3-2 treatment. (G)The MeRIP-PCR showed knockdown of METTL3 significantly decrease the m^{6}A modification level of BRD2. (H) The expression levels of BRD2 in 30 human GC tissues and 30 human normal tissues. (I) The expression level of BRD2 in 17 HP-positive and 17 HP-negative GC tumor tissues. (J) The IHC showed the expression level of BRD2 was highly expressed in HP-negative GC tissues than HP-positive GC tissues. (K) The IF of ASC after 5-FU and shBRD2 treatment. (L) The qRT-PCR tests on SS1-treated mice showed expression levels of METTL3 and BRD2. (M) The growth analysis of the organoid culture after shBRD2 and 5-FU treatment. (N) The ROS detection after treatment of shBRD2 and 5-FU in AGS and MKN-45. (U) The colony formation assay of AGS and MKN-45 cell lines after 5-FU and shBRD2. * $P<0.05$, ** $P<0.01$, *** $P<0.001$.

Figure S5

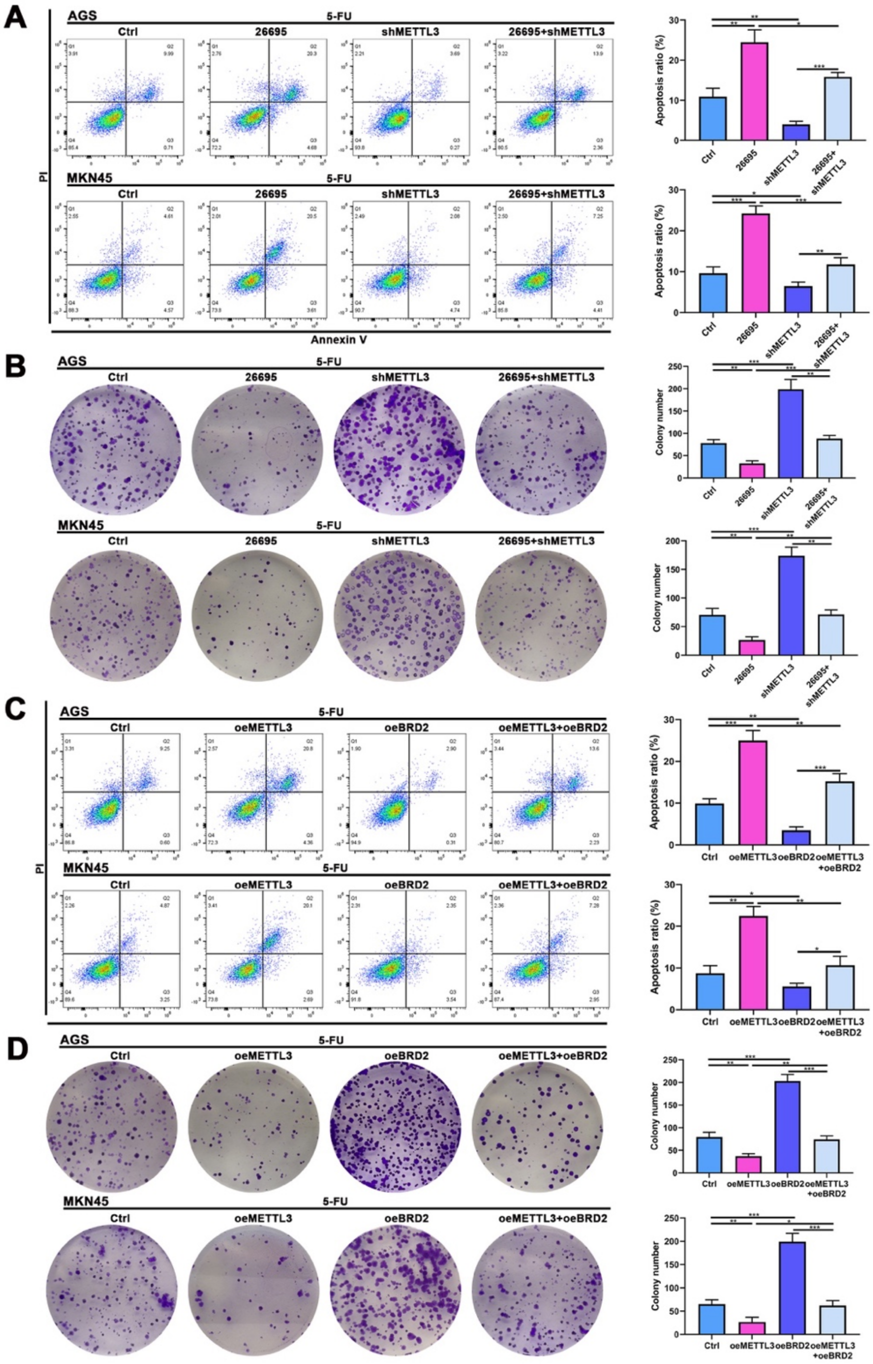

The rescue experiments confirmed that METTL3 could regulate apoptosis via BRD2

(A) The flow cytometry showed the apoptosis of AGS and MKN-45 after treatment of shMETTL3, HP infection and 5-FU (20 μM). (B) The colony formation assay analysis of AGS and MKN-45 after treatment of shMETTL3, HP infection and 5-FU. (C) The flow cytometry showed the apoptosis of AGS and MKN-45 after oeMETTL3, oeBRD2, and 5-FU. (D) The colony formation assay analysis of AGS and MKN-45 after treatment of oeMETTL3, oeBRD2, and 5-FU. * P<0.05, ** P<0.01, *** P<0.001.

Figure S6

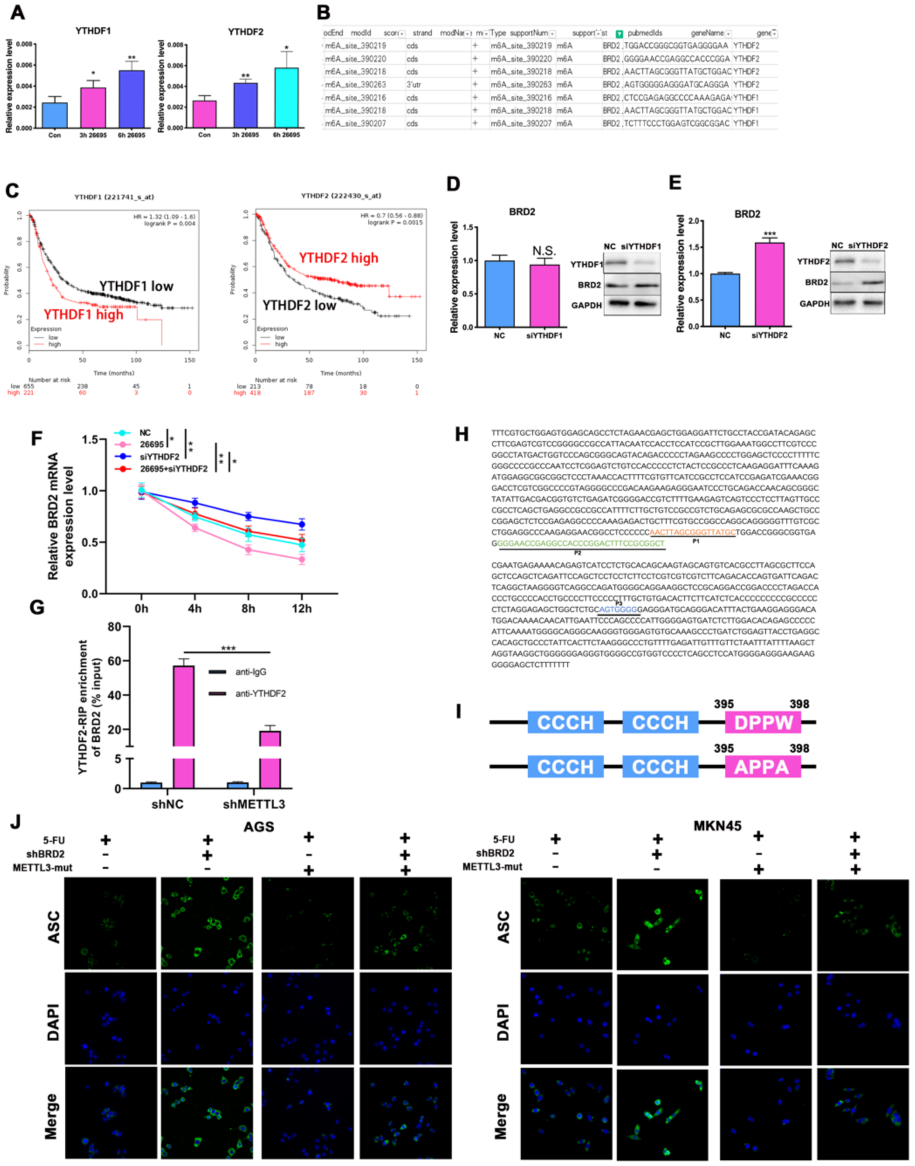

The prediction and binding capacity of YTHDF2 to BRD2

(A) The expression levels of both YTHDF1 and YTHDF2 after 3h and 6h of HP infection by qRT-PCR. (B) The RMBase prediction analysis of recognizer protein of BRD2. (C) The TCGA data showed prognosis data of YTHDF1 and YTHDF2. (D) The expression of BRD2 after knockdown of YTHDF1. (E) The expression of BRD2 after knockdown of YTHDF2. (F) The half-life of BRD2 after HP infection and siYTHDF2 treatment. (G) The RIP assay showed the capacity of YTHDF2 binding to BRD2. (H) The predicted binding sites with P1, P2 and P3 of BRD2 and YTHDF2 by Jaspar. (I) The key point mutations (D395A and W398A) of METTL3. (J) The IF of ASC in AGS and MKN-45 cell lines after 5-FU, shBRD2 and METTL3-mut treatment. *, P<0.05, **, P<0.01, ***, P<0.001.

Figure S7

A

C5	Complement C5	P01031	0	0.01861	0.01516	0.12392	0.10625	0.11854	0.01126	0.11624	10.3258	UP	0.00017	YES
FOXO4	Leucine-rich alpha-2-glycoprotein	P02750	0	0	0	0.7405	0.49017	0.66482	0	0.63183	0	UP	0.00104	YES
ANXA1	Annexin A1	P04083	11.7835	14.2919	16.8731	0.17914	0.51452	0.14975	14.3162	0.28114	-50.922	DOWN	0.00068	YES
HRG	Histidine-rich glycoprotein	P04196	0	0.01635	0	0.05235	0.07014	0.04353	0.00545	0.05534	10.1527	UP	0.00638	YES
ENO1	Alpha-enolase	P06733	60.3257	65.9249	64.512	29.9008	32.7522	25.221	63.5875	29.2913	-2.1709	DOWN	0.00024	YES

B

Matrix ID	Name	Score	Relative sc	Sequence	Start	End	Strand	Predicted sequence
MA0848.1	FOXO4	12.7136	1	seq1	1669	1675	+	GTAAACA
MA0848.1	FOXO4	8.82526	0.923128	seq1	1585	1591	+	GAAAACA
MA0848.1	FOXO4	8.5719	0.918119	seq1	1273	1279	+	ATAAATA
MA0848.1	FOXO4	7.14871	0.889983	seq1	451	457	+	AAAAACA
MA0848.1	FOXO4	7.14871	0.889983	seq1	1595	1601	+	AAAAACA
MA0848.1	FOXO4	7.14871	0.889983	seq1	1605	1611	+	AAAAACA
MA0848.1	FOXO4	7.14871	0.889983	seq1	1985	1991	+	AAAAACA
MA0848.1	FOXO4	6.74742	0.88205	seq1	1659	1665	+	GACAACA
MA0848.1	FOXO4	6.0096	0.867463	seq1	1611	1617	+	ATACACA
MA0848.1	FOXO4	5.33769	0.854179	seq1	1794	1800	+	CTAAACA
MA0848.1	FOXO4	5.22102	0.851873	seq1	590	596	+	GTACATA
MA0848.1	FOXO4	5.19794	0.851417	seq1	576	582	+	GTAATCA
MA0848.1	FOXO4	5.10649	0.849609	seq1	455	461	+	ACAAACA
MA0848.1	FOXO4	5.10649	0.849609	seq1	1599	1605	+	ACAAACA
MA0848.1	FOXO4	4.915	0.845823	seq1	1277	1283	+	ATAAAAA
MA0848.1	FOXO4	4.7052	0.841675	seq1	1381	1387	+	GCCAACA
MA0848.1	FOXO4	4.7052	0.841675	seq1	1770	1776	+	GCCAACA
MA0848.1	FOXO4	4.68358	0.841248	seq1	1409	1415	+	AAAAATA
MA0848.1	FOXO4	3.95179	0.82678	seq1	1747	1753	+	GTCAAGA
MA0848.1	FOXO4	3.53676	0.818575	seq1	708	714	+	GTGAATA
MA0848.1	FOXO4	3.02864	0.80853	seq1	514	520	+	ACCAACA
MA0848.1	FOXO4	2.70323	0.802097	seq1	544	550	+	GAAAAAA
MA0848.1	FOXO4	2.70323	0.802097	seq1	1978	1984	+	GAAAAAA

C

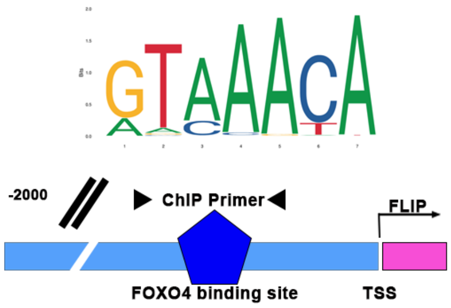

The COIP-MS analysis of BRD2 and prediction of FOX4

(A) The COIP-MS analysis of BRD2 contained FOXO4, the potential transcriptional factor of FLIP. (B) The potential transcription factor FOXO4 was predicted to bind with FLIP by Jaspar. (C) The motif of binding site of FOXO4 and promoter sequence of FLIP.

Supplementary Table. 1 Antibodies used in this study

Primary antibody		
Total Gasdermin D	Abcam	ab209845
Cleaved Gasdermin D	Abcam	ab215203
Total PARP	Cell signaling Technology	9532
Cleaved PARP	Abcam	ab32064
METTL3	Cell signaling Technology	86132
YTHDF1	Cell signaling Technology	86463
YTHDF2	Proteintech	24744-1-AP
P65	Cell signaling Technology	8242
p-P65	Cell signaling Technology	3033
BRD2	Abcam	ab245436
FOXO4	Abcam	EPR5442
FLIP	Abcam	ab8421
Caspase8	Abcam	ab32397
Cleaved Caspase8	Cell signaling Technology	8592
GAPDH	Cell signaling Technology	97166
Secondary antibody		
Anti-rabbit IgG	Cell signaling Technology	7074
Anti-mouse IgG	Cell signaling Technology	7076

Supplementary Table. 2 Sequences of primers used in this study

Primer sequence	
β-actin	Forward: 5'-ATTGCCGACAGGATGCAGAA-3'<br>Reverse: 5'-GCTGATCCACATCTGCTGGAA-3
METTL3	Forward: 5'-GAAAGACTATCTCCTGGCACTC-3'<br>Reverse: 5'-GTACCTTTGCTTGAACCGTG-3'
BRD2	Forward: 5'-TCGCATGGCCATTCCGGCAG-3'<br>Reverse: 5'-AAGGGGCTGGGCTGGAGGAG-3'
YTHDF1	Forward: 5'-CAAGCACACAACCTCCATCTTCG-3'<br>Reverse: 5'-GTAAGAAACTGGTTCGCCCTCAT-3'
YTHDF2	Forward: 5'-TAGCCAGCTACAAGCACACCAC-3'<br>Reverse: 5'-CAACCGTTGCTGCAGTCTGTGT-3'
FLIP	Forward: 5'-TTACACAGGCAGAGGCAAGA-3'<br>Reverse: 5'-GCTGGACTGGGTGTACTTCT-3'
METTL3-1-CHIP	Forward: 5'-GCATCTCAGTGTCACCTTACCT-3'<br>Reverse: 5'-TTCCCAGATAATTTCCTACAGCAT-3'<br>(Primer1: -1664~-1152 bp)
METTL3-2-CHIP	Forward: 5'-CCCACTGTTCTTATATTTTCAAGGT-3'<br>Reverse: 5'-TGTCTTAAACAAATAGGTTCCCAGA-3'<br>(Primer2: -1147~-1065 bp)
METTL3-3-CHIP	Forward: 5'-GTGTATGTCTTTTTCTCTCTTAGGG-3'<br>Reverse: 5'-GGCCTCTGCTGAAGGAAGAA-3'<br>(Primer3: -855~-620 bp)
BRD2-1-CHIP	Forward: 5'-GTCTGTCCACCCCCTCTACT-3'<br>Reverse: 5'-GTCTGTCCACCCCCTCTACT-3'
BRD2-2-CHIP	Forward: 5'- TTAGCGGGTTATGCTGGACC-3'<br>Reverse: 5'- AGCATCTTGACCGCAAGGAA-3'
BRD2-3-CHIP	Forward: 5'-AGGGCCCTGTTTTGAGATTGT-3'<br>Reverse: 5'- CCCCTTCTTCCCTCCCCAT-3'
